# Genome-wide analysis reveals molecular convergence underlying domestication in 7 bird and mammals

**DOI:** 10.1186/s12864-020-6613-1

**Published:** 2020-03-04

**Authors:** Yali Hou, Furong Qi, Xue Bai, Tong Ren, Xu Shen, Qin Chu, Xiquan Zhang, Xuemei Lu

**Affiliations:** 1 Key Laboratory of Genomic and Precision Medicine, Beijing Institute of Genomics, Chinese Academy of Sciences, Beijing, People’s Republic of China; 2 China National Center for Bioinformation, Beijing, People’s Republic of China; 3 University of Chinese Academy of Sciences, Beijing, People’s Republic of China; 4 Institute of Animal Husbandry and Veterinary Medicine, Beijing Academy of Agriculture and Forestry Sciences, Beijing, People’s Republic of China; 5 Guangdong Provincial Key Lab of Agro-Animal Genomics and Molecular Breeding, and Key Lab of Chicken Genetics, Breeding and Reproduction, Ministry of Agriculture, South China Agricultural University, Guangzhou, People’s Republic of China; 6 Center for Excellence in Animal Evolution and Genetics, Chinese Academy of Sciences, Kunming, People’s Republic of China

**Keywords:** Convergent evolution, Domestication and adaptation, Artificial selection, Behavioral modification

## Abstract

**Background:**

In response to ecological niche of domestication, domesticated mammals and birds developed adaptively phenotypic homoplasy in behavior modifications like fearlessness, altered sociability, exploration and cognition, which partly or indirectly result in consequences for economic productivity. Such independent adaptations provide an excellent model to investigate molecular mechanisms and patterns of evolutionary convergence driven by artificial selection.

**Results:**

First performing population genomic and brain transcriptional comparisons in 68 wild and domesticated chickens, we revealed evolutionary trajectories, genetic architectures and physiologic bases of adaptively behavioral alterations. To extensively decipher molecular convergence on behavioral changes thanks to domestication, we investigated selection signatures in hundreds of genomes and brain transcriptomes across chicken and 6 other domesticated mammals. Although no shared substitution was detected, a common enrichment of the adaptive mutations in regulatory sequences was observed, presenting significance to drive adaptations. Strong convergent pattern emerged at levels of gene, gene family, pathway and network. Genes implicated in neurotransmission, semaphorin, tectonic protein and modules regulating neuroplasticity were central focus of selection, supporting molecular repeatability of homoplastic behavior reshapes. Genes at nodal positions in trans-regulatory networks were preferably targeted. Consistent down-regulation of majority brain genes may be correlated with reduced brain size during domestication. Up-regulation of splicesome genes in chicken rather mammals highlights splicing as an efficient way to evolve since avian-specific genomic contraction of introns and intergenics. Genetic burden of domestication elicits a general hallmark. The commonly selected genes were relatively evolutionary conserved and associated with analogous neuropsychiatric disorders in human, revealing trade-off between adaption to life with human at the cost of neural changes affecting fitness in wild.

**Conclusions:**

After a comprehensive investigation on genomic diversity and evolutionary trajectories in chickens, we revealed basis, pattern and evolutionary significance of molecular convergence in domesticated bird and mammals, highlighted the genetic basis of a compromise on utmost adaptation to the lives with human at the cost of high risk of neurophysiological changes affecting animals’ fitness in wild.

## Background

Since the days of Darwin [1] it has been recognized that a succession of species tends to possess resembling modifications in behavior, morphology and physiology in response to domestication as compared with their wild ancestors, referred to as “domestication syndromes” [2]. It hitherto remains elusive how and when domestication originally started, but regardless, a diminished fear of humans is assumed to be a crucial prerequisite during the early phase of domestication. It has also been implicated, based on the experiments conducted on fox [3], rat [4] and chicken [5], that selection on reduced fearfulness initiates the acquisition of other domestication-related behavioral modifications by means of correlated responses, encompassing less aggression, elevated stress and social tolerances, altered activity, explorative tendency, and cognition, which partly or indirectly result in consequences for economic productivity [2, 5, 6]. One of the important aspects of further research in domestication is investigation of the basis for genetic selection that leads to tameness and other behavioral traits [7], which might reflect the mechanisms and consequences in shaping behavioral alteration during the early process of domestication. So far, the investigation has rarely been done. Only the tameness-related QTLs have been independently pinpointed in domesticated rat and fox based on genetic mapping [4, 8]. However, few orthologous regions have been identified.

Adaptively phenotypic homoplasy in divergent organisms may provide sources for identifying genetic architectures underlying complex traits. Domesticated animals share the set of adaptively behavioral alterations in response to human cohabitation, representing a typical convergent system and reasonably raising the key questions of whether molecular homoplasy exhibit and, if any, what are the characteristics and mechanisms, which remain largely undetermined. Repeated phenotypic adaptation was historically thought to occur primarily by evolution of divergent genetic mechanisms, whereas recent compelling evidences suggest that it is powerful enough to drive similar molecular mechanisms in independent lineages of the nature species [9–13]. So far, application of population genomic-based methods for detection of the genomic footprints under artificial selection has unraveled that genes associated with brain development in mammals have been often targeted [14–21], hinting towards a potential molecular similarity underlying parallel behavioral adaptations.

Parallelism of the molecular basis of convergent evolution may vary according to taxonomic scale of investigation and genetic distance of species [10, 12]. In addition to mammals, the traits in fear, aggression, activity level, explorative tendency and cognitive capability are also altered in domesticated birds [2, 22]. Such independent adaptations across genetically distant species provide an opportunity to comprehensively investigate the molecular parallelisms that may link to adaptive modification under domestication. So far, genome-wide scan for genetic basis of domestication hasn’t been implemented in all species like chicken. Although pioneering studies have been performed [23, 24], they are constrained by specific Tibetan chickens or pooled samples lacking individual-based genotype information to fully profile population architectures and genomic trajectories. In this study, we first carried out whole-genome re-sequencing and RNA sequencing (RNA-Seq) of 2 brain tissues from 6 representatively domesticated breeds and 3 wild red jungle fowl (RJF) populations, characterized the genomic footprints and brain transcriptional alterations thanks to domestication, revealed genetic bases of behavioral alterations. Integrating the genomic signatures targeted by positive selection in chicken and 6 other commonly domesticated farm or accompany mammals whose genome sequencing data or genome-wide mutations or the positively selected genes due to domestication are publically available, such as cattle, pig, rabbit, horse, dog, and cat, we further identified the convergent molecular signatures at hierarchical levels of mutation, gene, gene family, pathway and network, which were significantly involved in neuroplasticity, behavior modifications and human neuropsychiatric disorders. We verified a general hallmark of genetic burden in animal domestication. Our findings provide the first genomic evidence of convergent mechanisms underlying common neurophysiological/behavioral reshapes thanks to domestication across bird and mammals.

## Results

### Genetic diversity and introgression within and between wild and domesticated chicken breeds

Based on the fact that parallelism of molecular basis of convergent evolution may vary according to taxonomic scale of investigation and genetic distance of species [10, 12], we first characterized the genomic and transcriptional signature of domestication in chickens besides of mammals. A panel of 68 individuals from 3 RJF populations and 6 domesticated breeds that were not only specialized for meat production, egg laying, ornamental and medical purposes, but ranged from Chinese indigenous breeds to highly commercialized lines, were re-sequenced. An individual coverage of ~10X was achieved, followed by read mapping, variant calling and filtering. A total of 21,475,107 SNPs were identified, which was 1.88 times more than the SNPs that had been previously identified in pooled samples [23], and provided 61% more variants than those in the current variant repository (Ensembl Release 78). The details regarding samples and statistics of sequencing data and SNPs are summarized in Additional file 1: Table S1.

Understanding population architectures is fundamental for revelation of genetic basis under domestication. We first examined the genetic diversity (*π*), divergence and introgression within and between wild and domesticated chicken populations. The level of π was 1.2% in RJF, which was significantly higher than those (0.86% ± 0.04%) in domesticated breeds (*P* < 0.05), indicative of reduced genetic diversity during domestication. The π was the lowest in White Leghorn (WL) and Recessive White Rock (RW) (0.76% ± 0.03%), reflecting inbreeding of highly selected commercial strains with heightened egg and meat productions. Chinese indigenous breeds remained larger diversity (0.91% ± 0.07%).

**Fig. 1:**
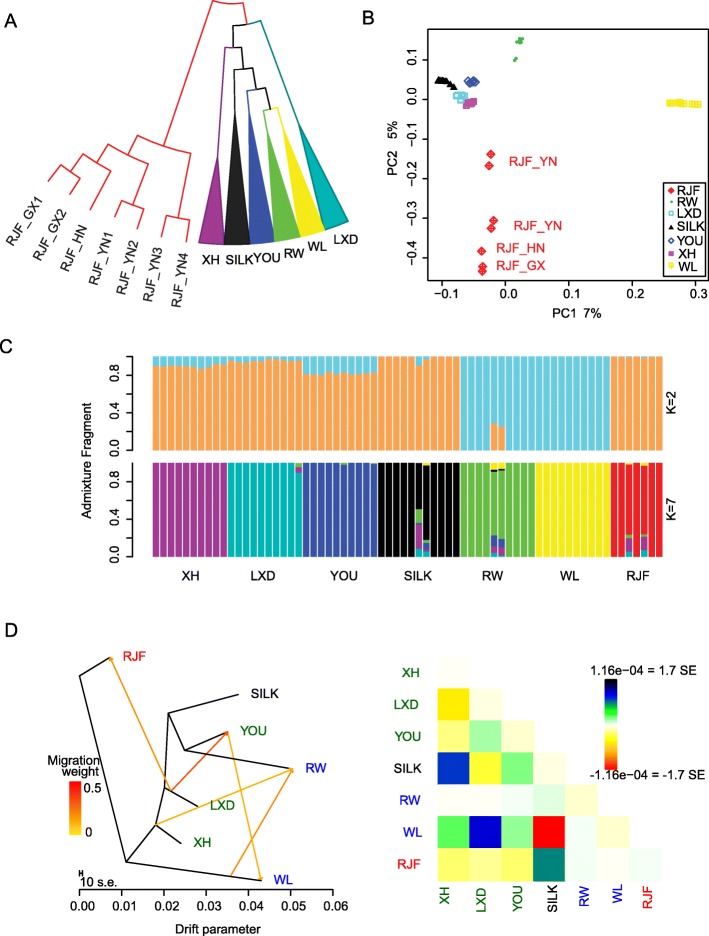
Population characteristics of wild and domesticated chickens. **a** Neighbor-joining phylogeny among wild and domesticated chickens based on genome-wide SNP data. The wild progenitors include red jungle fowls from the Guangxi (RJF_GX), Yunnan (RJF_YN) and Hainan (RJF_HN) provinces in China. The domesticated chickens consist of Chinese native breeds, including Guangdong Xinghua (XH), Luxi Dou (LXD), Beijing You (YOU), Silkie (SILK), and commercial strains such as Recessive White Rock (RW) and White Leghorn (WL). **b** The principle component analysis plot based on the first two principle components. **c** Population stratification and individual admixture with genetic cluster (K) equivalent to 2 and 7, respectively; colors in each column represent the individual ancestry proportions. **d** The maximum likelihood tree among chicken populations with 5 deduced introgression events and the residual matrix of the fitted model. The introgression events are highlighted as the arrows with colors from yellow to red, which represent the various weights of introgression (left panel). The arrow direction indicates the introgression direction. The residuals of the fitted model are illustrated in the right panel, where, the color in each cell [*i*, *j*] proportionally reflects the scaled residual covariance between population *i* and *j*, i.e. the residual covariance divides the average standard error (SE) of the observed covariances across pairs of population. The color scale bar is described in the palette on the right. Small residuals represent a well-fitted model. The fraction of the variance in relatedness among populations interpreted by the fitted model is 99.96%

Phylogenetic relationship and principle component analysis (PCA) congruously illustrated the divergence of the wild and domesticated chicken populations (Fig. 1). The RJFs separated from the domesticated breeds. The WL and RW first clustered together. Beijing You (YOU) and Silkie (SILK) were closer to the two commercialized breeds than other two Chinese native breeds, Xinghua (XH) and Luxi Dou (LXD). The top 2 components (PC1 and PC2), explaining 12% of the total variance, showed a strong correlation with chicken origin, breeding history and characteristic (Fig. 1b). According to PC1, the WL, a Europe-developed breed [25], showed larger difference from the Chinese native breeds than the RW. The genetic admixture of European and southeast Asian ancestors during the breeding of RW has been previously reported [26]. PC2 separated the domesticated chickens from the RJFs.

To interpret the population stratification and genetic admixture in wild and domesticated chickens, we performed a Bayesian clustering inference in ADMIXTURE that inferred the optimal number of genetic clusters to be *K* = 2 [27, 28]. We found that WL and RW belonged to one cluster, indicating the genetic differentiation from Chinese native breeds and RJFs due to origin rather than domestication (Fig. 1c). Slight genetic admixture was inferred by the other *K* values (Additional file 2: Figure S1). We used TreeMix [29] and a less-parameterized model of four-population test [30] to examine the topology of relationships and the history of genetic introgression among populations (Fig. 1d and Additional file 3: Figure S2, Additional file 1: Table S2). The inferred gene flow from LXD to RJF indicated the genetic introgression from domesticated to wild populations. Within the domesticated breeds, considerable gene flow between RW and WL (15~20%, *P* < 2.23e-308), between YOU and WL (13~33%, *P* < 5.66e-15), and between YOU and LXD (7~30%, *P* < 1.46e-09) was indicated, representing considerably genetic exchange or hybridization between distinct populations under intensive breeding programs for breed development and improvement.

### Selective sweeps and genes with selection signals in domesticated chickens

Population genomic analysis has revealed a complex genetic architecture in modern chickens. To identify genomic variations constrained by domestication rather than due to demography, introgression and specialization for various purposes, we examined selective sweeps and genes with selection signals in all domesticated breeds, which were not only used for varied purposes, but genetically divergent based on abovementioned results. We performed 5 different statistical methods, comprising the site frequency spectrum (SFS) - (heterozygosity, Tajima’s *D*, Fay & Wu’s *H* and SweepFinder) and linkage disequilibrium (LD) - based methods (integrated haplotype score, *iHS*). To be conservative, genomic regions that were supported by at least two of the tests were identified as sweeps and used in the following analysis (Additional file 4: Figure S3).

A total of 281 selective sweeps were identified on autosomes, representing 2% of the genome. Within the sweeps, 244 of 390 Ensembl genes presented significant signals of selection in their gene body or promoter regions. Based on the Ingenuity pathway analysis (IPA), the 244 positively selected genes (PSGs) exhibited an over-representation on the biological categories of nervous system function, ophthalmic disease, developmental disorder, energy production and immunological disease (Additional file 1: Table S3), reflecting the reshapes of the nervous system, metabolism and immune system under selective regimen of domestication.

The conspicuous signals of selection were related to the nervous system which is the most enriched term of the networks. According to IPA, ~ 30% of the PSGs (65/216) were associated with the developmental processes of nervous system and brain, including genesis, projection, branching and extension of neurons, neurotransmission, and synaptic plasticity (*P* < 0.001, Additional file 1: Table S4). Re-analyzing the expression pattern of these genes across 6 tissues based on the published RNA-Seq data in RJFs [31], we found that 92% of the 65 genes were exclusively or predominantly expressed in cerebrum and cerebellum (Additional file 5: Figure S4). Intriguingly, behavioral features involved by these genes interrogated fear response, anxiety, exploratory and hyperactive behaviors, locomotion, learning, cognition and conditioning (*P* < 0.001, Additional file 1: Table S4), which congruently matched the observed behavioral modifications under animal domestication, i.e. reduced fear and anxiety, decreased explorative tendency, altered activity, locomotion, learning and memory capability [2, 5, 22].

### Gene expression changes in brain tissues of domesticated chickens compared to RJFs

The analysis of the selective sweep suggests that neurological functions and related behavioral alterations might be the main targets of selection during the early phase of chicken domestication. Performing RNA-Seq for 2 brain tissues of 11 domesticated chickens from 4 modern breeds and downloading the published RNA-Seq data in 2 RJFs [31] (Additional file 1: Table S5), we detected the gene expression difference in brain tissues between wild and domesticated chickens to investigate whether the PSGs may play a role in the gene expression changes, resulting in the traits related to brain functions and behavioral alterations in domesticated chickens.

Gene expression profiles of cerebrum and cerebellum in domesticated individuals clustered together, unambiguously separating from RJFs (Additional file 6: Figure S5). One thousand six hundred eighteen and eight hundred forty two differentially expressed genes (DEGs) in cerebrum and cerebellum between wild and domesticated samples (*P*_*adj*_ < 0.01 and fold change > 1.5) were detected. According to the gene ontology analysis, neurological processes in which the DEGs were significantly enriched (*P* < 0.01, Additional file 1: Table S6), were congruent with the functional classifications of PSGs. Intriguingly, genes associated with the functions of cerebrum and cerebellum, such as neurological system processes and sensory (visual and auditory) perception, were consistently down-regulated in domesticated chickens. This may imply substantial functional alteration of brains in domesticated samples, or be correlated to their decreased brain size relative to body size [32]. In contrast, genes participating in RNA splicing and translation were significantly up-regulated in domesticated animals, indicative of significance of post-transcriptional regulation during the short-term evolutionary process.

**Fig. 2:**
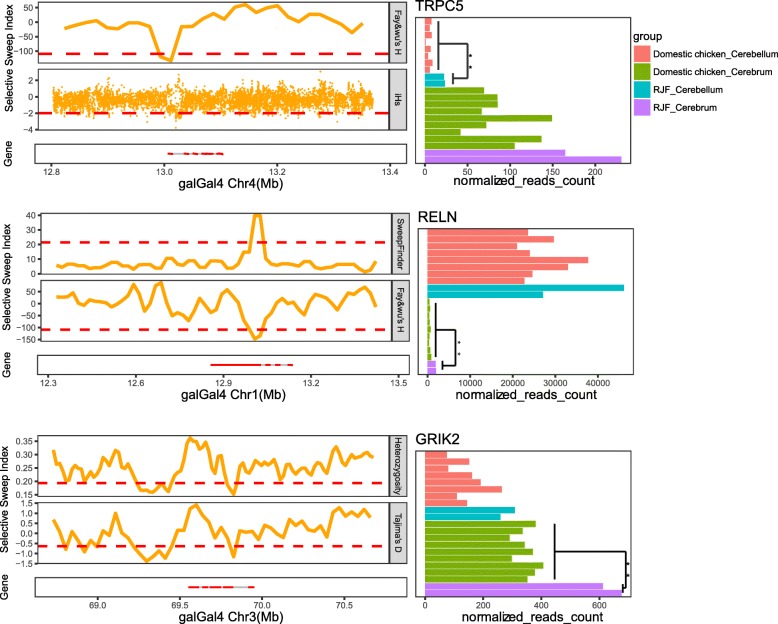
Selection signal and expression profiles of three representative genes associated with behavioral modification during domestication. These genes are both positively selected genes and differentially expressed genes in cerebrum or cerebellum in domesticated chickens, whose functions in domestication-related behavioral alterations have been verified in mutant mouse models. The left panels indicate statistics across different methods including Heterozygosity, Tajima’s *D*, Fay and Wu’s *H*, integrated haplotype score (*iHS*) and SweepFinder. The vertical axes represent the statistical values for each method; the horizontal axes represent the genomic coordinates around the target genes. The red dashed line in each method denotes the threshold above/below which the signals were considered as positive selection. The right panels illustrate the gene expression levels of cerebrum and cerebellum in wild and domesticated chickens, where the double asterisks represent significant difference with a *P* value < 0.01

Up to 22% (53/244) of the PSGs displayed expression difference in brain tissues between wild and domesticated chickens, being a set of genes adapted to domestication at both genomic and transcriptional levels. These genes possess the potential of altering neurological functions according to their enrichment in the processes of neuritogenesis, synaptic transmission, neuron and neuroglia development, and long-term potentiation (*P* < 0.05, Additional file 1: Table S7). Eight of these genes have been verified to be responsible for behavioral changes in mutant mice, consisting of fear response (*GRIK2*, *TRPC5*), social interaction (*TRPC5*), learning and memory (*NF1*, *RELN*, *CSMD1*, *AQP4*), exploration and locomotion (*ERC2*, *OMG*), which have been regarded as the consequent behavioral phenotypes of chicken domestication [33–40] (Fig. 2 and Additional file 7: Figure S6).

To further investigate the potentially regulatory effects of the 53 PSGs on gene expression changes, we searched for the interactive genes of the PSGs in a chicken protein-protein interaction network (https://string-db.org/). Thirty of the 53 PSGs were present as part of the network. By implementing Wilcoxon rank-sum test, the 30 PSGs tend to be hub nodes in comparison with 30 randomly selected genes (*P* < 10 ^− 8^), supporting the genes at nodal positions in regulatory networks are preferred targets of evolutionary changes [41]. In addition, the 30 PSGs interacted with 15% more DEGs compared to other DEGs (Chi-square test, *P* = 0.018), suggesting the expression changes of these PSGs more likely resulted in regulation alteration of the DEGs in brain. Functionally, these PSGs primarily regulate the DEGs through calcium signaling, long-term potentiation and depression, CREB and nNOS signaling in neurons, axonal guidance and glutamate receptor signaling (Additional file 8: Figure S7). All these findings propose that PSGs play a vital role in regulating gene expression changes that may result in the neurological alterations and behavioral modifications that underlie chicken domestication.

### Common targets of selection involved in behavior modification among domesticated species

Modification in behavior is believed to be a key aspect of the early phase in animal domestication [2, 22]. Several previous studies have identified various genomic regions with selection signals that might be associated with domestication-related behavioral traits in different mammals [14–20]. In this study, the genes that we identified under positive selection are also involved in the neurological processes and resembling behavioral traits in chicken. Given the similarity of the consequences of domestication, it raises whether there are unique genomic properties on which strong selection prefers to act in all domesticated animals. To answer it, we profiled the genomic signals of selection and transcriptional modifications if available in 7 domesticated species to decipher the potential targets that commonly appear in a variety of animals.

Using the same methods and PSG filtering criteria that we used in chicken, we identified the selective sweeps and PSGs based on the SNPs called from whole-genome sequence data in 55 dogs and 7 wolves from DoGSD (http://dogsd.big.ac.cn), and 100 domesticated and 34 wild pigs in Asia and Europe from PigVar (http://res.xaut.edu.cn/pigsps). In total, we detected 206 and 353 PSGs in dogs and pigs, respectively. Since the sequence data and the identified SNPs in cat, cattle, horse and rabbit cannot be accessed in public database, we directly used the list of selected genes that have been reported in the literatures for these mammals [16, 18, 20, 21]. Totally, 291, 83, 101 and 100 PSGs, from cat, cattle, horse and rabbit, respectively, were identified.

According to IPA for PSGs in each of the species, we found that the network of nervous system development and function was commonly enriched in all domesticated animals (Additional file 1: Table S8), suggesting that evolution of parallel molecular mechanisms might lead to the phenotypic convergence in nervous system and behaviors in these divergent animals. Evidences that parallel evolution may result from common variants at specific nucleotide sites, in homologous genes, pathways, and networks have been reported in many taxa [9–13]. Therefore, we integrated the PSGs involved in nervous system functions (Additional file 1: Table S9) to search for common molecular mechanisms at multiple levels: mutations, genes, gene families, pathways and networks.

#### Mutations and genes

No shared amino acid substitution was observed across domesticated species. None PSG was shared by at least 4 species. Twenty six genes were recurrently detected as PSGs in 2 domesticated species, and 1 in 3 species (Additional file 1: Table S10), whose orthology across species was confirmed by performing synteny analysis (Additional file 1: Table S11). *TCTN3*, the only gene detected in 3 species that encodes tectonic protein, functions in neural tube patterning, and causes holoprosencephaly and neural tube defects that are the most common risks of anomalies in the central nervous system [42]. IPA showed that the shared 27 genes appeared to be responsible for domestication-related behavioral aspects like fearlessness (*GRIK2*) and learning deficit (*NPAS3*) (*P* < 0.05, Additional file 1: Table S12).

#### Gene families

A total of 71 gene families were identified in more than one domesticated species. Based on IPA, top 2 enriched physiological processes of PSGs in these families were neurological functions and behaviors like anxiety, social exploration, cognition, exploratory behavior (*P* < 0.01, Additional file 1: Table S12). Based on Fisher’s exact test and FDR (false discovery rate) correction, 3 families including glutamate ionotropic receptors (GluIRs), semaphorins, and tectonic proteins, were significantly targeted by selection in at least 4 species (*P*_*adj*_ < 0.05, Additional file 1: Table S10, Additional file 9: Figure S8).

Genes encoding GluIRs were selected in 6 domesticated animals, including *GRIK2* in chicken and rabbit, *GRIK3* in cattle and dog, *GRIA1* in dog and cat, *GRIA2* in cat, and *GRID1* in horse. Semaphorin genes that act as axon guidance cues like *SEMA3A*, *SEMA3D*, *SEMA3E* and *SEMA3F*, were targeted in chicken, dog, and pig, respectively, while *SEMA6A* in cattle. Besides of the tectonic gene *TCTN3* that was simultaneously selected in 3 domesticated animals (chicken, dog, and pig), *TCTN1* presented signals of selection in horse.

Gene expression changes caused by mutations on these genes may be a major determinant of phenotypic variability. Supportably, our RNA-Seq data in cerebrum and cerebellum showed that expression of genes in these families like *GRIK2* and *SEMA3A* was significantly decreased in domesticated chickens than that in RJFs (Additional file 10: Figure S9). Re-analyzing the RNA-Seq data in frontal cortex from 3 pairs of Rongchang pigs and wild boars [43], and those in cerebellum and hypothalamus from pairs of 3 dogs and 1 wolf (personal communication), we observed a consistent decrease in gene expression of *GRIK2*, *SEMA3A*, *SEMA3D* and *SEMA3E* (declined by 0.10–0.86 fold) in any brain tissues in at least 2 domesticated animals although not all of them were significant DEGs (Additional file 10: Figure S9).

#### Pathways and networks

Selection may ultimately target the functional units of pathway and network [44]. To investigate if a set of PSGs as a whole to share function in common pathway or network, we mapped the PSGs relevant to neuronal functions onto KEGG (Kyoto encyclopedia of genes and genomes) pathways and performed Fisher’s exact test and FDR correction to identify the significantly enriched pathways by PSGs of species. A total of 38 enriched pathways were identified in 5 or 7 domesticated species (*P*_*adj*_ < 0.05, Additional file 1: Table S10), among which, 7 were related to neurotransmission and 8 were diverse signal transduction pathways.

**Fig. 3:**
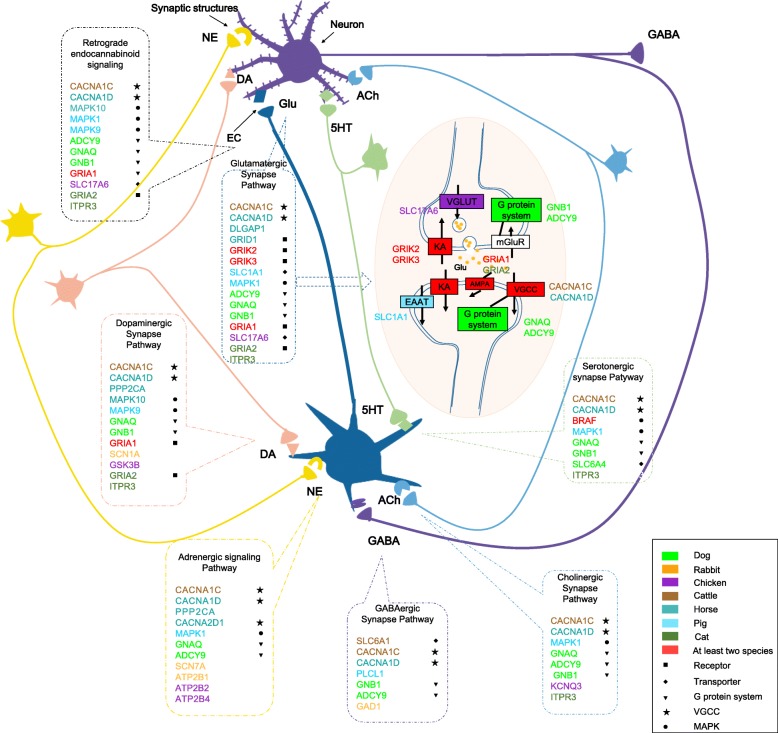
A conceptualized network for the 7 enriched neurotransmission pathways. The conceptualized figure is cited from the book of Stephen M. Stahl (2013). The 7 pathways involve transmissions of neurotransmitters of glutamate (Glu), (nor) epinephrine (NE), dopamine (DA), endocannabinoid (EC), γ-aminobutyric acid (GABA), serotonin (5HT) and acetylcholine (Ach) across presynaptic and postsynaptic neurons. The synaptic structures (pre- and post- synaptic terminals, and cleft) for each neurotransmitter signaling are marked on the neuron surfaces, around which, the positively selected genes (PSGs) within the signaling pathway are labeled with different colors for distinct species (chicken, dog, pig, cattle, horse and rabbit, cat). Glutamatergic synapse pathway is interpreted in detail in the middle, in which genes with signals of selection are marked

The 7 neurotransmission pathways involve transmissions of a variety of neurotransmitters of glutamate, (nor) epinephrine, dopamine, serotonin, endocannabinoid, γ-aminobutyric acid, and acetylcholine across presynaptic and postsynaptic neurons, which function in concert to build a complicated neuronal circuit in CNS [45]. According to adjust *P* values, glutamatergic synapse was the most significant neurotransmission pathway (*P*_*adj*_ = 4.56*10^− 7^), in which genes of GluIRs, voltage-gated calcium channels (VGCC), vesicular and postsynaptic transporters, and G-protein system presented significant signals of selection (Fig. 3). Within the 7 pathways, it was found that, adapted to domestication, although different genes tended to be positively selected in distinct species, there existed a repeated target on a limited set of genes encoding neurotransmitter receptors, transporters, G-protein system, VGCCs and mitogen-activated protein kinases (MAPKs), with proportions of the PSGs that involved these processes in each pathway as 54.54–91% (Fig. 3).

**Fig. 4:**
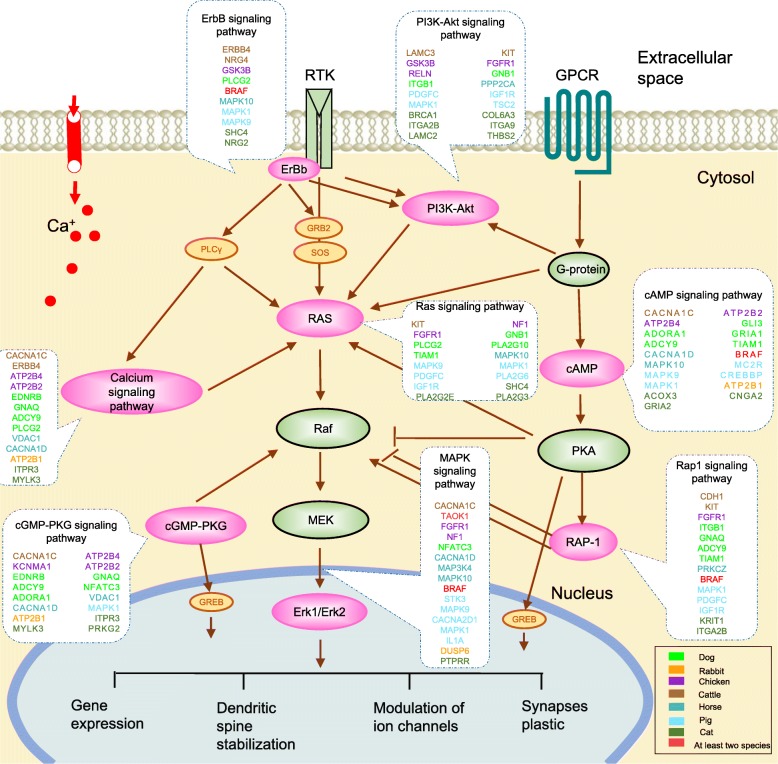
The interactive network for the 8 enriched signaling transduction pathways. The network was constructed according to the interaction relationships annotated in KEGG database. Each oval represents a signaling transduction pathway. The ovals with pink fillings represent the significantly shared pathways in 5–7 domesticated species. The positively selected genes (PSGs) within the signaling pathways are labeled around the ovals with different colors for distinct species. The red color indicates PSGs identified in at least 2 species. GPCR: G protein-coupled receptor; RTK: Receptor tyrosine kinase

During neurotransmission, neurotransmitters bind to synaptic receptors on pre−/post-synaptic neurons and result in long-term excitatory or inhibitory consequences through activation of a series of signal transduction cascades. The 8 signal transduction pathways that we identified involved cAMP, cGMP-PKG, MAPK, Ras, Rap1, ErbB, calcium and PI3K-Akt signaling. To reveal whether these pathways coordinate as signaling cascades in neurotransmission, we constructed their interactive network based on the interaction relationships annotated in KEGG database (Fig. 4). It was observed that these pathways substantially interplayed with each other, forming a network-based signaling cascade and ultimately converging on CREB and MEK/ERK systems to mediate expression of genes that may regulate functions of neurons and plastic changes necessary for domestication-related behaviors.

### Genetic burden of domestication across animals

It has recently been proposed as an intriguing evolutionary concept that there exists genetic cost of domestication in most plants [46, 47] and animals like dog [48–50] and horse [16]. It is reasonably wondering would it be a generally genetic burden on animal domestication. Herein, we systematically profile this feature across taxa of dog, pig and chicken.

Similar to dog [48–50], we first introduced human and zebra finch as outgroups of pig and chicken, respectively, based on which, we determined the allele states (ancestral or derived) of mutations in wild and domestic populations. We calculated the relative occurrence of nonsynonymous versus synonymous mutations (*d*_*N*_*/d*_*S*_ ratio or *ω*) for each individual. A significantly increased proportion of nonsynonymous SNPs (greater *ω*) was observed in domesticated chickens compared with the wild ancestors (ANOVA test, *P* < 0.01, Additional file 11: Figure S10), highlighting the cost of domestication, i.e. the excess accumulation of nonsynonymous mutants since domestication. The pattern was also observed in modern European pig compared with wild counterparts although the difference was not significant.

Further, based on a referred-to wild individual, we defined the allele states of mutations in domestic populations, and predicted the functional effect of nonsynonymous mutations using the GERP algorithm that assesses the position-specific estimates of evolutionary constraint using maximum likelihood evolutionary rate estimation [51]. It was excavated that 26.7, 25.3 and 20.5% of the nonsynonymous substitutions are predicted as functionally deleterious for pig, dog and chicken, respectively, by taking GERP score greater than 2 as applied in Henn et al. [52]. These deleterious variants consistently showed the evidence of domestication cost, with a significantly elevated proportion and frequency in domestic breeds in comparison with the wild progenitors (χ^2^ test, *P* < 0.005, Additional file 1: Table S13, Additional file 12: Figure S11). Furthermore, we unraveled the frequencies of deleterious mutation in domestic population present strong positive and negative correlations with LD magnitude (Pearson’s *r* = 0.80, *P* = 0.0564) and nucleotide diversity (Pearson’s *r* = − 0.90, *P* = 0.0158), respectively, indicative of reduced efficacy of purify selection.

### Common targets in domesticated species are evolutionary conserved and associated with neurological disorders in human

The convergent analysis among domesticated species indicates that genes and functional units relevant to neurological functions and behavior modifications have been identified under positive selection in both birds and mammals. Nevertheless, genes associated with brain functions are traditionally thought to be subjected to strong constraint and evolve slowly in mammals [53]. It is reasonably wondering what’s the functional significance or clinical implication for these commonly targeted PSGs. Evidence has suggested that there exist conserved molecular mechanisms between animal behaviors and human mental health disorders [54, 55]. We herein deduced that the common molecular targets of selection in different animals might be associated with neuropsychiatric disorders in human.

**Fig. 5:**
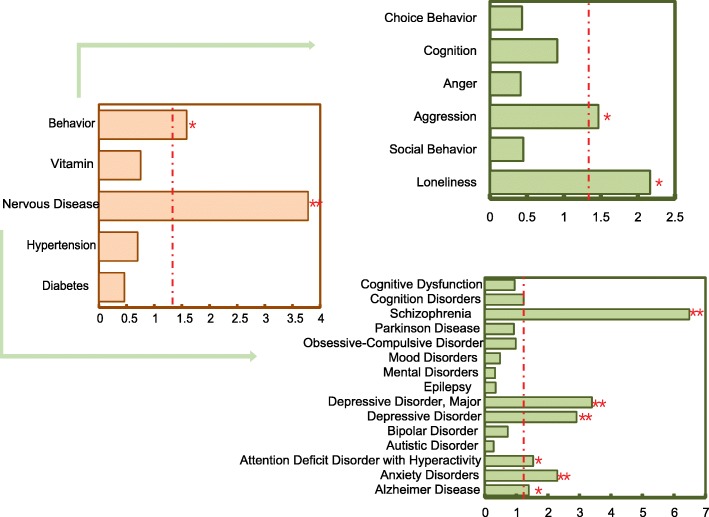
Enrichment analysis for the common PSGs in comparison with human PheGenI physiological traits. The nervous diseases and behaviors in human were included as tests, and the traits of hypertension, diabetes and vitamin were taken as controls. The horizontal axis represents the *P* value of enrichment analysis on an absolute logarithmic scale. The red dashed line indicates a *P* value cutoff of 0.05. One or two asterisk(s) indicate(s) the significant level of 0.05 or 0.01, respectively

We first defined the common molecular targets of positive selection as PSGs that showed signatures of selection in at least two species and were members in the common gene families and pathways that were shared by at least two species. We investigated degree of evolutionary conservation for the common PSGs. The phastCons and phyloP scores of the common PSGs among 100 vertebrates were significantly higher than those of background genes (Additional file 13: Figure S12), indicative of a high degree of conservation. We scanned the common targets against the vulnerability loci of various human physiological traits retrieved from genome-wide association studies from the PheGenI project [56], and revealed a significant enrichment of the common target genes in neurological diseases and behavioral disorders relative to control traits such as diabetes, hypertension, and vitamin traits (*P* < 0.05, Fig. 5, Additional file 1: Table S14). The overrepresented diseases and disorders by these common genes, such as anxiety, aggression, attention deficit hyperactivity, schizophrenia, depression and loneliness, displayed phenotypic analogy with domestication-related behavioral alterations. Apart from the genes that already existed in PheGenI database as putative risk factors of human neuropsychiatric diseases, the common target genes provided more novel candidates potentially related to the overrepresented diseases through functional interaction with the known risk factors in the same pathways (Additional file 14: Figure S13). The results suggest that domesticated animals could be natural disease models for understanding the role of genes implicated in neuropsychiatric disorders in human.

## Discussion

A fundamental question in evolutionary genetics concerns the extent to which parallel phenotypic adaptation in divergent organisms is attributed to convergent or parallel changes at molecular levels [12]. Recent evidence has suggested that the adaptively genetic evolution might be more predictable than was previously appreciated in nature species [9–13, 57–60]. However, basis and patterns of molecular homoplasy across domesticated species in response to intensified selection during a short period of time rather than nature selections in a long-term run remain largely elusive.

Parallelism of the molecular basis of convergent evolution may vary according to taxonomic scale of investigation and genetic distance of species [10, 12]. To investigate the general rule of adaptive convergence under domestication in divergent birds and mammals, we first profiled the signatures of chicken domestication at both genomic and transcriptional levels. Genetic diversity substantially reflects chicken breed origin, demography and specialization for various purposes. There exist massive introgressions from domesticated to wild chickens and among distinct breeds. Continuous gene flow to wild populations during or after domestication has also been reported in dog, pig and horse [61–63], whether the immigrant alleles are neutral, detrimental, or beneficial needs detailed spatio-temporal factors for further exploitation [64]. Considerable gene flow among breeds was attributed to intensive breeding programs for breed development and improvement. These results suggest introgressions need to be carefully considered when studying genetics and evolutions among wild and domesticated animals.

Consistent with domesticated mammals, chicken PSGs exhibit enrichment in neurological processes and brain functions, suggesting of parallel molecular mechanisms that may result in phenotypic convergence in behavior alterations. We have revealed novel genetic architectures and physiologic bases of behavioral alterations and vision degeneration during early phase of chicken domestication. Part of the identified PSGs have been suggested association with reduced fear, anxiety and explorative tendency, altered activity, locomotion, learning and memory capability using mouse model [33–40]. Intriguingly, genes relevant to brain functions in cerebrum and cerebellum were consistently down-regulated in domesticated chickens, which may imply substantial functional alteration, or be correlated to their decreased brain size relative to body size [32]. Consistently, knockout of *SLC2A1*, a down-regulated gene, has been verified to be associated with neurodegeneration, behavioral deficits and microcephaly, i.e. abnormally small head in mice [65]. Similar pattern was also observed in dog, where majority of DEGs (80.44–92.82%) were down-regulated in comparison with wolf across brain tissues of cerebellum and hypothalamus. These results may be correlated to the fact that domesticated animals of the same species evolved significantly reduced brain sizes compared to their wild relatives, relative to body size, in the process of adaptation to ecological niche of domestication [32]. Contrastly, although domesticated chickens were also found to have relatively reduced brain mass compared to RJF, they underwent an absolutely enlarged cerebellum independent of increased body size that was supposed to occur in light of domestication [66]. Due to the complexity, genetic mechanism by which domestication affects brain size or composition warrants further investigation. In contrast, genes participating in RNA splicing, e.g. the spliceosome snRNA or proteins and serine/arginine proteins, were significantly up-regulated, highlighting significance of post-transcriptional mechanism, i.e. RNA splicing, in response to chicken domestication. Whereas, this pattern is ambiguous in dog and pig by additionally re-analyzing the RNA-Seq data in frontal cortex from pairs of Rongchang pigs and wild boars [43], which may be attributed to the fact that avian genomes experienced extensive shorting of introns and reduced intergenic distances compared to mammals [67] and alternative splicing might be the mostly efficient mechanism to increase proteome diversity to adapt to evolutionary transition.

Of note, from the perspective of network for the PSGs in chicken, we have observed that the genes at nodal positions in a *trans*-regulatory network are preferred targets of domestication. To test whether this is also applicable to other domesticated animals, we scrutinized the PSGs in dog and pig, respectively. By performing Wilcoxon rank-sum test, the PSGs tend to be hub nodes in comparison with randomly selected genes with comparable gene numbers (*P* < 10 ^− 8^, Additional file 15: Figure S14), supporting the hypothesis that genes at nodal positions in regulatory networks are favored targets of evolutionary changes [41].

By integrating the selection signals in the genomes of chicken and 6 domesticated mammals, we profiled the genetic basis and patterns of parallel evolution at different hierarchical levels. No common amino acid substitution is observed, which indicates adaptively convergent phenotypes may not always arise from the same substitution, revealing a potential role of contingency in the context of epistatic interactions in divergent species [10, 68]. In contrast, mutations in adaptation to domestication presented a convergent enrichment in non-coding regions in chicken, pig and dog (Additional file 16: Figure S15), where abundant *cis*-regulatory elements reside. These support that mutations in regulatory sequences may be important for driving phenotypic adaptation in response to domestication. Non-coding region like introns have been studied as essential mediators of cell response to starvation [69].

A convergence of domestication occurs at the level of gene and gene family. A limited gene set, especially those encoding GluIRs, semaphorins and tectonic proteins, was repeatedly targeted across species. GluIRs respond to glutamate via receptor-type-dependent neurotransmission, regulating neuronal excitability and synaptic plasticity. *GRIK2* and *GRIA1* have been confirmed as molecular basis of neurodegeneration and domestication-related behavioral alterations like fearfulness, anxiety, social exploration, learning and memory [34, 70]. *GRIK3* and *GRIA1* have also been identified as overlapped genes under selection in domesticated dogs, cattle, horses and cats [71]. Semaphorins act as axon guidance molecules by specifying cell morphology or inducing cell collapse as a result of change in cytoskeleton and cell adhesion, mediating neural circuit developments including, but not limited to, neurite extension, dendrite specification, axon sorting and synaptic specificity [72]. Association of *SEMA3F* with fear and *SEMA6A* with exploration and learning has been reported in knockout mice [73, 74]. Tectonic protein gene *TCTN3* functions in neural tube patterning and exhibits holoprosencephaly and neural tube defects in the mutant mice [42]. Intriguingly, we have noticed that five shared positively selected genes or gene family members, including *BRAF*, *SEMA3A*, *SEMA3F*, *SEMA3D*, and *MAPK1*, are known to be involved in neural crest cell development and migration [75, 76], which appears to support the neural crest hypothesis of domestication syndrome [77].

A striking common pattern targeting pathways of neurotransmission and signal transduction cascades was uncovered across species, among which glutamatergic synapse is the mostly shared. Both neurotransmissions and signal transductions are known to regulate synaptic plasticity and neural circuits that underlie emotional, social and cognitive function, in which many genes have been demonstrated association with domestication-related behavioral alterations such as reduced fear and explorative tendency, altered learning and memory capability in a variety of studies in knockout mice [78–81]. Although different genes within the same pathway tend to be selected in distinct species, there exists a repeated target on genes encoding neurotransmitter receptor, transporter, G-protein system, VGCC, MAPK and signaling cascades. GluIR and ERK/MAPK signaling pathways has previously been highlighted in multiple domesticated animals [71]. These results highlight that domesticated species repeatedly evolved similar behavioral adaptions through certain molecular repeatability at the levels of pathways and networks, but having more than one solution, i.e. diverse selected genes in distinct species within the same pathway. In spite of molecular convergence, different domesticated animals experienced significantly varied domestication routes, for instances, dog, cat, chicken and pig for the commensal pathway with diverged purposes, cattle and rabbit for the prey pathway, and horse for the directed pathway [82]. Besides, changes in social ecology of animals (i.e., both feeding niche and social organization), along with other parameters such as interaction into environments, have also been recently suggested to contribute to domestication process [83]. How different routes of domestication give rise to different biological processes will be targeted in the future study.

The predominant factor accounting for the genetic burden is rationally attributed to the Hill-Robertson effect that selection on linked sites hinders the purging of deleterious variants and impinges the overall effectiveness of selection in finite populations in the absence of recombination [84]. Intensive artificial selection on beneficial sites during domestication and breed improvement decreases locus-specific *Ne*, thereby increases the retain probability of linked deleterious mutations due to hitchhiking, and hinders their efficient purging under purifying selection [46, 48, 49, 84]. Demographic history such as severe bottlenecks during domestication and breed formation that leads to relaxation of purifying selection on slightly pernicious mutations also contributes to the accumulation [47, 48]. Summarily, accumulation of detrimental mutations is widely acknowledged as the genetic load of domestication across a wide variety of domestic species, eliciting a general hallmark of domestication.

The commonly selected genes associated with domestication-related behavioral alterations were a set of conserved genes, which benefitted the fitness of species, i.e. ability to survive and reproduce in wild environment [85]. By contrast, these genes that experienced positive selection during domestication may give rise to high fitness of domesticated animals under circumstances of human beings. The adaptive changes caused by these genes in reduced fear, aggression and anxiety, increased social tolerance, altered activity and cognition, were all preferable for successful domestication [2, 5, 6]. The commonly selected genes displayed enrichment in analogous neuropsychiatric disorders in human, consisting of anxiety, aggression, attention deficit hyperactivity, schizophrenia, depression and loneliness, and provided potential novel risk factors underlying these diseases. Reports on couple positively selected genes in multiple domesticated species that are strong candidates for neurodevelopmental diseases and syndromes support the finding [71]. These results render domesticated animals as natural disease models in understanding the roles of genes implicated in neurodegeneration and neuropsychiatric disorders in human.

## Conclusions

In this paper, we provide one of the genome-wide views of the evolutionary trajectories, including population hierarchy and introgressions, among 6 domesticated chicken breeds and 3 wild RJF populations. Our study reveals strong signatures of selection around genes associated with the nervous system and behavior modification, similar to mammals adapted to domestication. We have systematically recapitulated the molecular mechanisms of parallel evolution underlying behavior modifications across 7 domesticated species, and reveal an appreciated genetic bias during the evolution. We disclose the repertoire of convergent selection molecules that used to be constrained by purify selection has experienced positive selection after transitions from wild to domesticated constraints, and propose conservation between these genes and risk factors underlying human neuropsychiatric disorders. These findings implicate a compromise between utmost adaptation to human cohabitation and higher risks of neurophysiological changes that influence the fitness of the animals in wild. Our analyses provide valuable theories for the underlying convergent evolution across birds and mammals.

## Methods

### Chicken breeds, samples, and tissues

A total of 68 individuals from 7 typical populations composed of 4 Chinese indigenous breeds (XH from the Guangdong province; LXD from the Shandong province; YOU from Beijing; SILK from the Jiangxi province), 2 highly commercialized breeds (RW and WL), and 3 wild red jungle fowl populations (RJF) were investigated (Additional file 1: Table S1). For each breed, 6 males and 4 females were sampled for whole-genome sequencing (WGS). The blood samples were taken from the wing vein by the standard venepuncture procedure. Chickens were kept alive after blood collection. A Taiwan Silkie with accession number of PRJNA202483 was also included [86]. For RJF, 1 male and female from the Guangxi province were sampled for WGS. In addition, 3 males and 2 females from the Yunnan and Hainan provinces with accession number PRJNA241474 were obtained [87].

Eleven adult chickens were anesthetized with phenobarbital sodium solution (30 mg/kg) and immediately euthanized by rapid decapitation. Seventeen cerebrum and cerebellum samples from eleven LXD, SILK, RW, and WL males were dissected and collected for RNA-Seq (Additional file 1: Table S5). Additionally, 4 transcriptomes of cerebrum and cerebellum for RJFs (One male and one female for each tissue) with accession numbers SRR306710–306713 were also analyzed [31].

### DNA extraction, library construction, and WGS

Genomic DNA was extracted from blood with QIAamp® DNA Mini Kit (QIAGEN, Duesseldorf, Germany). DNA quality and concentration were monitored using agarose gel electrophoresis, Bioanalyzer 2100 system (Agilent Technologies, CA, USA), and Qubit® 2.0 Fluorometer (Life Technologies, CA, USA). Sequencing libraries were constructed by following IlluminaTruSeq™ DNA preparation kit, and sequenced on Hiseq2000 platform. Approximately 92~140 millions of clean paired-end reads with a length of 100 bp were generated for each individual (Additional file 1: Table S1).

### RNA extraction, library construction, and RNA-Seq

Total RNA was extracted from tissues using TRIzol reagent (Invitrogen, CA, USA). RNA quality and concentration were determined using NanoPhotometer® spectrophotometer (Implen, CA, USA), Qubit® 2.0 Fluorometer (Life Technologies, CA, USA), agarose gel electrophoresis, and Bioanalyzer 2100 system (Agilent Technologies, CA, USA). Sequencing libraries with poly(A) enrichment were constructed by following IlluminaTruSeq™ RNA sample preparation kit, and sequenced on Hiseq2500 platform, achieving an average of 21.86 million clean paired-end reads with a length of 125 bp for each sample (Additional file 1: Table S5).

### Detection of genomic variations

Clean paired-end reads were aligned to the indexed chicken reference genome (galGal4) by running the maximal exact matches algorithm in BWA [88]. After alignment, multi-sample calling based on Bayesian inference algorithm [89] was implemented to identify and genotype SNPs using SAMtools and BCFtools [90]. PCR duplicates were removed by applying rmdup. Mpileup was used to pile up the base-pair alignment information for reads, and -C50 was used to downgrade the mapping quality for reads containing excessive mismatches. A site was considered to be a SNP when the following post-filters were applicable: 1) read depth less than 100; 2) base quality score > =20 and mapping quality score > =10; 3) variant occurred with a probability of 99%, i.e., the variant/reference quality score > =20; 4) alternative allele supporting reads > = 3.

### Calculation of genetic diversity and introgression within and between chicken populations

The genetic diversity (π) was calculated as normalized SNP number by the genome size, which was compared across populations using ANOVA and Shaffer’s multiple comparison tests.

Neighbor-joining tree was constructed using PHYLIP [91] based on 1,809,179 independent SNPs. SNPs in strong LD, i.e., having *r*^*2*^ > 0.1 with neighboring SNPs, were excluded using a 100-SNP sliding window (stepped by 20 SNPs) implemented in PLINK [92]. Genetic divergence among chicken populations was investigated using PCA in EIGENSOFT program [93] based on all SNPs.

Population stratification and genetic admixture in wild and domesticated chickens were inferred based on independent SNPs by implementing ADMIXTURE [27]. Various genetic clusters (*K*) (ranging from 2 to 7) were applied. The optimal *K* was determined through a cross validation procedure [28].

Introgression was derived using TreeMix [29]. Maximum likelihood bifurcating tree was constructed based on the independent SNPs. Introgression events were subsequently interrogated using “-m 8” based on all SNPs, where TreeMix accounted for LD by grouping SNPs in a window size (−*k*) of 100,000, corresponding to 6.26 Mb, which was comparable to the window size (10 Mb) that was used for humans [94]. The fraction of variance that can be interpreted by the fitted model was calculated as described in Pickrell and Pritchard (2012) [29].

Four-population test was executed in TreeMix to verify the derived introgression events. The *f*_*4*_ (A, B; C, D) was calculated as described in Reich et al. (2009) [30], and its standard error (SE) was estimated using a Block Jackknife procedure with a block size of 100,000 SNPs. To assess significance of introgression, Z-score was calculated as $\frac{f4\left(\mathrm{A},\mathrm{B};\mathrm{C},\mathrm{D}\right)}{SE_{f4\left(\mathrm{A},\mathrm{B};\mathrm{C},\mathrm{D}\right)}}$, which normally distributed with mean = 0 and variance = 1 under the null hypothesis [30]. Significantly positive Z-score (Z-score > 4) indicates gene flow between A and C or between B and D, whereas, negative (Z-score < − 4) implies introgression between A and D or between B and C [95].

### Identification of putative selective sweeps (PSS) and genes with selection signals

Based on previous studies [15, 17, 23], we determined the window size for selective sweep screening as 40 kb for chicken and 100 kb for pig and dog, which far exceed the LD extent to avoid confusion between selection and demography. Window-sliding step was set as half of the window size.

Heterozygosity (*H*_*p*_) [23] was calculated as *H*_*p*_ = 2 ∑ *n*_max_ ∑ *n*_min_/(∑*n*_max_ +  ∑ *n*_min_)^2^, where *n*_max_ and *n*_max_ are numbers of the most and least alleles within populations at a specific SNP, and ∑ is the sum of SNPs within windows. Threshold to claim a PSS was determined based on a randomly shuffled method [96]. Genome was split into 1-kb windows with estimates of ∑*n*_max_ and ∑*n*_min_, which were randomly shuffled for 1000 times to disturb LD. For each shuffled genome, we calculated *H*_*p*_ within windows and recorded the minimum. This resulted in a set of minimums with a dimension 1000. We chose the 50th lowest value as cutoff, which indicated a significant level of 0.05.

Tajima’s *D* [97] was deduced as $\frac{{\hat{\theta}}_{\pi }-{\hat{\theta}}_w}{\sqrt{\hat{Var}\left({\hat{\theta}}_{\pi }-{\hat{\theta}}_w\right)}}$, where ${\hat{\theta}}_{\pi }$ indicates the average heterozygosity within populations, equivalent to $\sum \limits_{i=1}^{n-1}\frac{2{S}_ii\left(n-i\right)}{n\left(n-1\right)}$; *S*_*i*_ is the number of derived variants found *i* times in a sample of *n* chromosomes; ${\hat{\theta}}_w$ is derived from the number of segregating sites equal to ${\left(\sum \limits_{i=1}^{n-1}\frac{1}{i}\right)}^{-1}\sum \limits_{i=1}^{n-1}{S}_i$. The calculation was implemented using VCFtools [98]. In contrast, Fay & Wu’s *H* [99] was calculated as $\frac{{\hat{\theta}}_{\pi }-{\hat{\theta}}_H}{\sqrt{\hat{Var}\left({\hat{\theta}}_{\pi }-{\hat{\theta}}_H\right)}}$, where ${\hat{\theta}}_H=\sum \limits_{i=1}^{n-1}\frac{2{S}_i{i}^2}{n\left(n-1\right)}$. The deduction was performed using VariScan [100]. For both statistics, the bottommost 1% windows were considered as PSSs.

Haplotypes were constructed using fastPHASE [101] based on SNPs with minor allele frequency > 5%. *iHS* [102] for each core SNP was derived as $\frac{\mathit{\ln}\left(\frac{iHH_A}{iHH_D}\right)-{E}_p\left[\mathit{\ln}\left(\frac{iHH_A}{iHH_D}\right)\right]}{SD_p\left[\mathit{\ln}\left(\frac{iHH_A}{iHH_D}\right)\right]}$, where *iHH*_*A*_ indicates the integral of decay of extended haplotype homozygosity (*EHH*) away from the ancestral allele until EHH reaches 0.05; *iHH*_*D*_ is the integrated *EHH* with respect to the derived allele; *E*_*p*_() and *SD*_*p*_() are the expectation and standard deviation of $\mathit{\ln}\left(\frac{iHH_A}{iHH_D}\right)$, estimated from the empirical distribution at SNPs whose derived allele frequencies (*p*) match that of the core SNP. Further, a statistic of a window was defined as proportion of SNP with *iHS <* − 2 [102, 103]. The corresponding empirical *P* value was assigned as follows [103]: 1) binned the windows by similar SNP numbers with an increment of 10 and 2) for each bin, the empirical *P* value for a specific window was assigned as the fraction of windows owning relatively larger statistics. During the process, the bins with windows less than 20 were removed. The windows with the lowest 1% of the empirical *P* value were considered as PSSs.

SweepFinder was implemented to determine PSSs based on spatial pattern of SFS around the focal SNP and the algorithm of composite likelihood ratio (CLR), where null hypothesis is neutral SFS pattern and alternative hypothesis allows selection, conferring robustness to complex demography and recombination rate [104]. Grid size was assigned as number of SNPs on each chromosome since the method is single point based [105]. For the focal SNP, surrounding sites were taken into account with a cut-off of *αd* = 12, where *d* is the distance to the focal SNP; *α* = *rln*(2*N*)/*s*; *N* is the population size; *r* is the recombination rate per base pair; *s* is the selection coefficient. The cut-off corresponds to a probability of escaping a sweep of 0.999994 for surrounding SNPs. The maximum of CLR within a window was presented as the statistic. Eventually, the windows with the topmost 1% of the statistics were considered as PSSs.

Final selective sweeps were ascertained as the conserved set that were identified by at least two of the methods so as to limit method-specific bias. Additionally, selective sweeps do not necessarily mean that the overlapped genes have selection signals. We further filtered the genes with selection signals using the following procedures: 1) for each method, with the exception of *iHS*, we calculated 1-kb window statistics within the selective sweeps. The 1-kb windows with 2% bottommost statistics (topmost for SweepFinder) that were assumed to have selection signals were selected, while the windows with SNPs less than 2 were excluded; 2) the sweep-overlapped genes were eliminated when they or their promoter regions (2 kb upstream gene start site) were barely supported by any of the selected 1 kb-windows; 3) since the selected 1 kb-windows for Tajima’s *D* and heterozygosity are bias to have less SNPs (Additional file 4: Figure S3B), the genes with selection signals were required to encompass at least 10 selected 1-kb windows to avoid Type I errors, where the filter strategy of 10 was determined based on the frequency of selected 1-kb windows that supported the genes (Additional file 4: Figure S3C); and 4) the genes containing any SNP with *iHS* < − 2 were included since *iHS* considers not only the focal SNP but also the surrounding mutations. Specifically, the pigs in Asia and Europe were independently analyzed to account for distinct origins and the final PSGs were combined.

### Gene expression pattern of the 65 PSGs related to neurodevelopment

Gene expression pattern of the 65 PSGs is based on the published RNA-Seq data of cerebrum, cerebellum, testis, liver, kidney and heart in RJF with accession number SRR306710-SRR306713 [31]. The expression level for each gene was characterized by reads per kilobase of exon model per million mapped reads (*RPKM*), and was calculated based on the G_values provided by Brawand et al. [31]. Formula for calculation referred to Qi et al. [106].

### Identification of differentially expressed genes

Clean paired-end reads from RNA-Seq data for each individual were aligned to the reference genome using TopHat v2.0.13 [107] with default settings. The normalized count value for each gene was calculated by the relative log expression method of DESeq [108] based on raw count provided by HTSeq [109]. The phylogenetic relationships among transcriptomes were profiled using pheatmap under R environment with a distance algorithm of Pearson correlation and a hierarchy method of complete Hclust. DEGs between groups were identified using DESeq with filters of *P*_*adj*_ < 0.01 and fold change (FC) > 1.5.

Six transcriptomes of frontal cortex from 3 pairs of Rongchang pigs and wild boars with accession numbers SRR5470021~5,470,026 [43] were downloaded and re-analyzed.

### Determining the predisposition to being hub nodes for the 30 PSGs

A chicken protein-protein interaction network was accessed from STRING database (version 10.5, https://string-db.org/), encompassing 7108 genes and 399,271 interactions if interrogating interaction types of activation, binding, catalysis, inhibition, post-translational modification, and reaction. To determine whether the 30 PSGs are predisposed to being hub nodes in the network, we compared the degrees of the 30 genes and those of 30 randomly selected genes in corresponding sub-networks. Degree of a gene was defined as number of interactions that the gene had with the other genes in the network. For the 30 PSGs, we extracted a sub-interaction network, comprising 1800 genes and 3166 interactions. Within the sub-network, we calculated degrees and degree ranks of the 30 PSGs. For comparison, we randomly selected 30 genes for 1000 times. For each time, we extracted the sub-network and calculated the degree ranks of these genes. The median degree ranks of the 1000 samplings were assigned as those for 30 randomly selected genes. We compared the ranks of the 30 PSGs and those of 30 randomly selected genes using Wilcoxon rank-sum test. The PSGs presenting a significantly higher rank of degree were considered to be predisposed to being hub nodes.

### Definition of gene family, pathway, and network for PSGs

We intersected the PSGs with gene family that was approved by HUGO gene nomenclature committee and curated in an online repository (https://www.genenames.org). We mapped the PSGs onto the annotated KEGG pathways (https://www.genome.jp/kegg/pathway.html). A high degree of conservation in the functional properties of proteins encoded by orthologous genes across domesticated species and human, especially in the context of pathway, is assumed based on the previous evidence and the feature of protein-sequence similarity [110, 111]. Fisher’s exact tests and false discovery rate corrections of Bonferroni correction were applied to test the enrichment of the PSGs in annotated gene families or pathways in HUGO and KEGG datasets. We constructed the network of 8 signal transduction cascades by interrogating interaction relationships in KEGG pathway database.

### Association of the commonly targeted genes of positive selection with human neuropsychiatric disorders

We downloaded the human Phenotype-Genotype dataset from PheGenI project [56]. The commonly targeted genes by positive selection were compared to the vulnerability loci of human nervous system diseases (including Alzheimer Disease, Autism Spectrum Disorder, Bipolar Disorder and other 19 terms) and behaviors (including Loneliness, Aggression, Cognition and other 12 terms), with those of Hypertension, Diabetes, and Vitamin as controls. Details of the human PheGenI physiological traits are presented in Additional file 1: Table S14. Fisher’s exact test was applied to determine the enrichment of the commonly targeted genes on the vulnerability genes of human physiological traits.

### Enrichment analysis of the putatively adaptive mutations

We defined SNPs within sweeps with *iHS* < − 2 in domesticated animals as putatively adaptive mutations in response to domestication. We downloaded gene annotation via ANNOVAR (Wang et al., 2010), and calculated the percentages of exon, intron, upstream, downstream, UTR3, UTR5, splicing and intergenic regions across selective sweeps. Upstream and downstream regions were defined as 1 kb upstream and downstream the transcription start and end site, respectively. For each region, SNPs with *iHS* < − 2 were counted as observed number, and the expected number was calculated as the total observed number multiplied by the percentages of this region. Based on the observed and expected numbers for each region, significant level was determined by Chi-square test and further corrected by Bonferroni adjustment.

### Calculation of *d*_*N*_/*d*_*S*_ ratios in wild and domestic populations across species

We utilized zebra finch as outgroup to unbiasedly deduce the allele states (ancestral or derived) of mutations in wild and domestic chickens. We firstly downloaded the chained BlastZ pairwise alignments of zebra finch (taeGut2) and chicken genomes (galGal4) from the University of California, Santa Cruz Genome Browser (http://hgdownload.soe.ucsc.edu/goldenPath/galGal4/ vsTaeGut2/). The alignments were further trimmed into protein-coding regions according to the chicken genome annotation (galGal4), comprising a total of 17,041 Ensembl genes and 23.43 Mb in length (1% of the total). The identified SNPs for each chicken were then assigned onto the trimmed alignments, where the allele states were exquisitely determined. When a site represented distinguished alleles between wild and domestic chickens and one of those matched the zebra finch sequence, the base from zebra finch was considered as ancestral state. Cases where a site was endowed with at least three types of alleles across zebra finch, wild and domestic chickens were ignored. The trimmed alignments were concatenated and further analyzed using CODEML in PAML to calculate the *d*_*N*_/*d*_*S*_ ratio in wild and domestic chickens. ANOVA was implemented for population comparison. Similar processes were implemented for pig by using outgroup of human.

### Identification of deleterious nonsynonymous mutations

According to the well-clustered wild individuals and domesticated individuals, as well as the substantial genetic divergences between them, principally, any of the wild sample could be used to define the allele states of mutations in domestic populations despite of few improperly determined mutations. For chicken, the reference genome is indeed a wild bird, which was used to infer the allele states of mutations. For dog, we used LUPWCHN00001 (Grey wolf, Haerbin, China) as the reference. For pig, SRS387323 (Wild boar-2) was used as reference of Asian Pig, and WB25U11 (WB25U11_WildBoar) of European pig. We computed the genomic evolutionary rate profiling (GERP) scores using the GERP++ code for all nonsynonymous SNPs, which reflect whether an amino acid substitution is likely to significantly affect protein function based on sequence homology and physical property of amino acids. We downloaded FASTA alignments of 45 vertebrate genomes aligned to hg19 (http://hgdownload.cse.ucsc.edu/goldenPath/hg19/multiz46way/alignments/), and ran each exon of gene through GERP++, omitting the dog and pig sequence, respectively, where the phylogenetic tree “46way.nh” was used. We then converted the CanMap3.1 coordinates of dog SNPs and susScr3 coordinates of pig SNPs to hg19 coordinates using LiftOver to annotate them with a GERP score. Sites with > 2 rejected substitution score per site were considered deleterious.

## Supplementary information

**Additional file 1.** Supplementary Tables

**Additional file 2: Figure S1.** The inferred population stratification and individual genetic admixture in wild and domesticated chickens. A. The cross-validation procedure to infer the optimal number of genetic clusters (*K*) that presents the minimum cross-validation error. B. The delivered population stratification and individual genetic admixture with varying *K* (ranging from 2 to 7); colors in each column represent the individual ancestry proportions. XH: Guangdong Xinghua, LXD: Luxi Dou, YOU: Beijing You, SILK: Silkie, RW: Recessive White Rock, WL: White Leghorn, RJF: Red jungle fowls.

**Additional file 3: Figure S2.** The deduced introgression events among chicken populations. RJFs are integrated into one outgroup in A and B, and are subdivided into distinct populations based on their geographic locations (RJF_HN from the Hainan province, RJF_GX from Guangxi, RJF_YN from Yunnan, among which, RJF_YN_mix indicates two admixed individuals) in C and D. A and C. (Left panel) The maximum likelihood tree (the breed symbol in red color represents red jungle fowls, blue: the commercial breeds, green: Chinese native breeds, black: the Silkie) and (Right panel) the residual matrix of the fitted model, where, the color in each cell [*i*, *j*] proportionally reflects the scaled residual covariance between population *i* and *j*, i.e. the residual covariance divides the average standard error (SE) of the observed covariances across pairs of population. The color scale bar is described in the palette on the right. Residuals above zero represent populations that are more closely related to each other in the data than in the fitted tree, and thus are candidates for introgression events. The fitted tree accounts for 87.43% of the variance in relatedness among populations in A, and 98.50% in C. B and D. (Left panel) The maximum likelihood tree with 5 and 8 deduced introgression events and the residual matrix of the improved model. The introgression events are highlighted as the arrows with colors from yellow to red, which represent the various weights of introgression. The arrow direction indicates the introgression direction. (Right panel) The residuals of the improved model are illustrated, where the color scale is the same as that in A. The fraction of the variance in relatedness among populations interpreted by the improved model rises up to 99.96% in B and 99.97% in D.

**Additional file 4: Figure S3**. The selective sweeps derived from multiple statistics and the filtering strategy for the positively selected genes. A. Venn diagram of the identified selective sweeps from five methods including Heterozygosity, Tajima’s *D*, Fay and Wu’s *H*, integrated haplotype score (*iHS*) and SweepFinder. B. The density of SNPs with selection signals in 1-kb windows that locate in the identified selective sweeps and have 2% bottommost/topmost statistics for each method, reflecting the selection bias against smaller SNP number for Tajima’s *D* and Heterozygosity. C. The frequency of selected 1-kb windows supporting selective sweep genes for each method, determining the filter strategy of selective sweep genes as 10 1 kb-windows for Tajima’s *D* and Heterozygosity.

**Additional file 5: Figure S4.** The expression pattern across 6 chicken tissues for the 65 selective sweep genes related to neurodevelopment. The gene expression pattern is based on the published RNA-Seq data of RJFs [31]. The gene expression level is calibrated on the logarithmic scale. The tissues include cerebrum, cerebellum, testis, liver, kidney and heart. For each tissue, one male and one female were examined, with the exception of testis where two males were examined.

**Additional file 6: Figure S5.** Clustering analyses of gene expression in cerebrum and cerebellum across chicken populations.

**Additional file 7: Figure S6.** Selection signal and expression profiles of five representative genes associated with behavioral modification during chicken domestication. These genes are both positively selected genes and differentially expressed genes in cerebrum or cerebellum in domesticated chickens, whose functions in domestication-related behavioral alterations have been verified in mutant mouse models. The left panels indicate the statistics across different methods including Heterozygosity, Tajima’s *D*, Fay and Wu’s *H*, integrated haplotype score (*iHS*) and SweepFinder. The vertical axes represent the statistical values for each method; the horizontal axes represent the genomic coordinates around the target genes. The red dashed line in each method denotes the threshold above/below which signals were considered as positive selection. The right panels illustrate the gene expression levels of cerebrum and cerebellum in wild and domesticated chickens, where the double asterisks represent significant difference with a *P* value < 0.01.

**Additional file 8: Figure S7.** The regulatory network between the 53 positively selected genes (PSGs) and the differentially expressed genes (DEGs) of cerebrum and cerebellum in domesticated chickens compared with their wild counterparts. The molecules filled by orange color indicate the overlapped genes between PSGs and DEGs. The molecules filled by red or green color represent the up- or down-regulated DEGs, respectively, where the shades of filled colors represent the extent of the alteration at gene expression level. The solid lines imply direct interactions between molecules. The ovals filled by yellow color at outer layer indicate the canonical pathways related to neurological functions, in which, both PSGs and DEGs are known to participate.

**Additional file 9: Figure S8**. Selective signals on genes in convergent family of glutamate ionotropic receptors, semaphorins and tectonic proteins. The species include chicken (galGal), dog (canFam) and pig (susScr). The methods encompass Heterozygosity, Tajima’s *D*, and Fay and Wu’s *H*, integrated haplotype score (*iHS*) and SweepFinder. The vertical axes represent the statistical values for each method; the horizontal axes represent the genomic coordinates around the target genes. The red dashed line in each method denotes the threshold, above/below which signals were considered as positive selection. The red bars in genes indicate the exon regions of gene.

**Additional file 10: Figure S9.** Expression level of genes in convergent family of glutamate ionotropic receptors, semaphorins and tectonic proteins in paired wild and domesticated animals. The species include chicken (galGal), dog (canFam) and pig (susScr). Brain tissues include cerebrum and cerebellum for chicken, frontal cortex for pig, and cerebellum and hypothalamus for dog. The vertical axes represent the gene expression levels; the horizontal axes indicate the genes in families of glutamate ionotropic receptors, semaphorins and tectonic proteins.

**Additional file 11: Figure S10.** The comparison of nonsynonymous versus synonymous mutations (d_N_/d_S_ ratio) between wild and domestic breeds across chicken **(A), pig (B) and dog (C).** Chicken population includes XH (Guangdong Xinghua), LXD (Luxi Dou), YOU (Beijing You), SILK (Silkie), RW (Recessive White Rock), WL (White Leghorn) and RJF (Red jungle fowls). One-way ANOVA is used for the significance test.

**Additional file 12: Figure S11**. The comparison of frequency of deleterious variants between wild and domestic breeds across pig, dog and chicken.

**Additional file 13: Figure S12.** Conservation degree of the commonly targeted genes of selection. The cumulative distributions of phastCons and phyloP scores for the commonly targeted genes of selection (red) in comparison with other background genes (blue). Wilcoxon rank-sum test was implemented to check the significance of difference.

**Additional file 14: Figure S13.** Comparison of the commonly selected genes in domesticated species with the risk factors of enriched human neurological diseases and behavioral disorders annotated in the PheGenI project. The commonly selected genes, were defined as those that showed signatures of selection in at least 2 domesticated species and were members in the common gene families and pathways that were shared by at least two species. The enriched neurological diseases and behavioral disorders in humans were anxiety, aggression, attention deficit hyperactivity, schizophrenia, depression and loneliness. The risk factors of these diseases were obtained from the PheGenI project. The conserved genes between the commonly selected genes during domestication and the risk factors of human neurological diseases and behavioral disorders were marked by circles and filled with different colors for different species. Red color indicates genes selected in at least 2 species. Besides of the conserved genes, other commonly selected genes may provide more risk factors (grey color) that may serve as novel candidates for these diseases, which were potentially related to the overrepresented neuropsychiatric diseases through functional interaction with the existed risk factors in the same pathways.

**Additional file 15: Figure S14**. The rank of degree of the PSGs and the randomly selected genes from dog and pig gene-gene interaction network. The orange line is the rank of degree of the PSGs, calculated from their subnetwork and sorted by increasing x-coordinate. The blue line is the median rank of degree of randomly selected genes (Equal number with PSGs) in their subnetwork after 1000 times sampling.

**Additional file 16: Figure S15.** Enrichment of the putatively adaptive mutations in different genomic regions across pig, dog and chicken. The putatively adaptive mutations in response to domestication were defined as SNPs within selective sweeps with iHS < − 2 in domesticated animals. Genomic regions of exon, intron, upstream, downstream, UTR3, UTR5, splicing, and intergenic regions were considered. Upstream and downstream regions were defined as 1 kb upstream and downstream the transcription start and end site, respectively. The pigs in Asia and Europe were independently analyzed to account for their distinct origins. The significantly enriched genomic regions were circumscribed. Double asterisks represent significant difference with a *P* value < 0.01.

## Data Availability

The 62 genome sequencing data and 17 transcriptome data in chicken have been deposited in the archive of Beijing Institute of Genomics (https://bigd.big.ac.cn/gsa/) under the accession number CRA000005.

## Abbreviations

CLR: Composite likelihood ratio
DEG: Differentially expressed gene
FDR: False discovery rate
GERP: Genomic evolutionary rate profiling
H_p_: Heterozygosity
IPA: Ingenuity pathway analysis
KEGG: Kyoto encyclopedia of genes and genomes
LD: Linkage disequilibrium
LXD: Luxi Dou chicken
PCA: Principle component analysis
PheGenI: Phenotype-Genotype Integrator
PSG: Positively selected genes
PSS: Putative selective sweeps
QTL: Quantitative trait loci
RJF: Red jungle fowl
RW: Recessive white rock chicken
SE: Standard error
SFS: Site frequency spectrum
SILK: Silkie chicken
WGS: Whole-genome sequencing
WL: White Leghorn chicken
XH: Xinghua chicken
YOU: Beijing You chicken
