# Supplementary figures and images for: Genome-wide analysis reveals molecular convergence underlying domestication in 7 bird and mammals
Source: BMC Genomics. 2020 Mar 4;21:204. doi: 10.1186/s12864-020-6613-1 (PMC7057487; doi:10.1186/s12864-020-6613-1)

**A**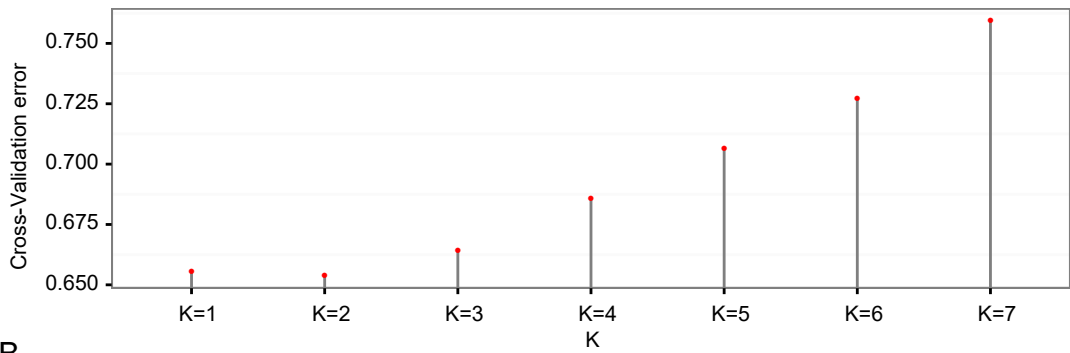**B**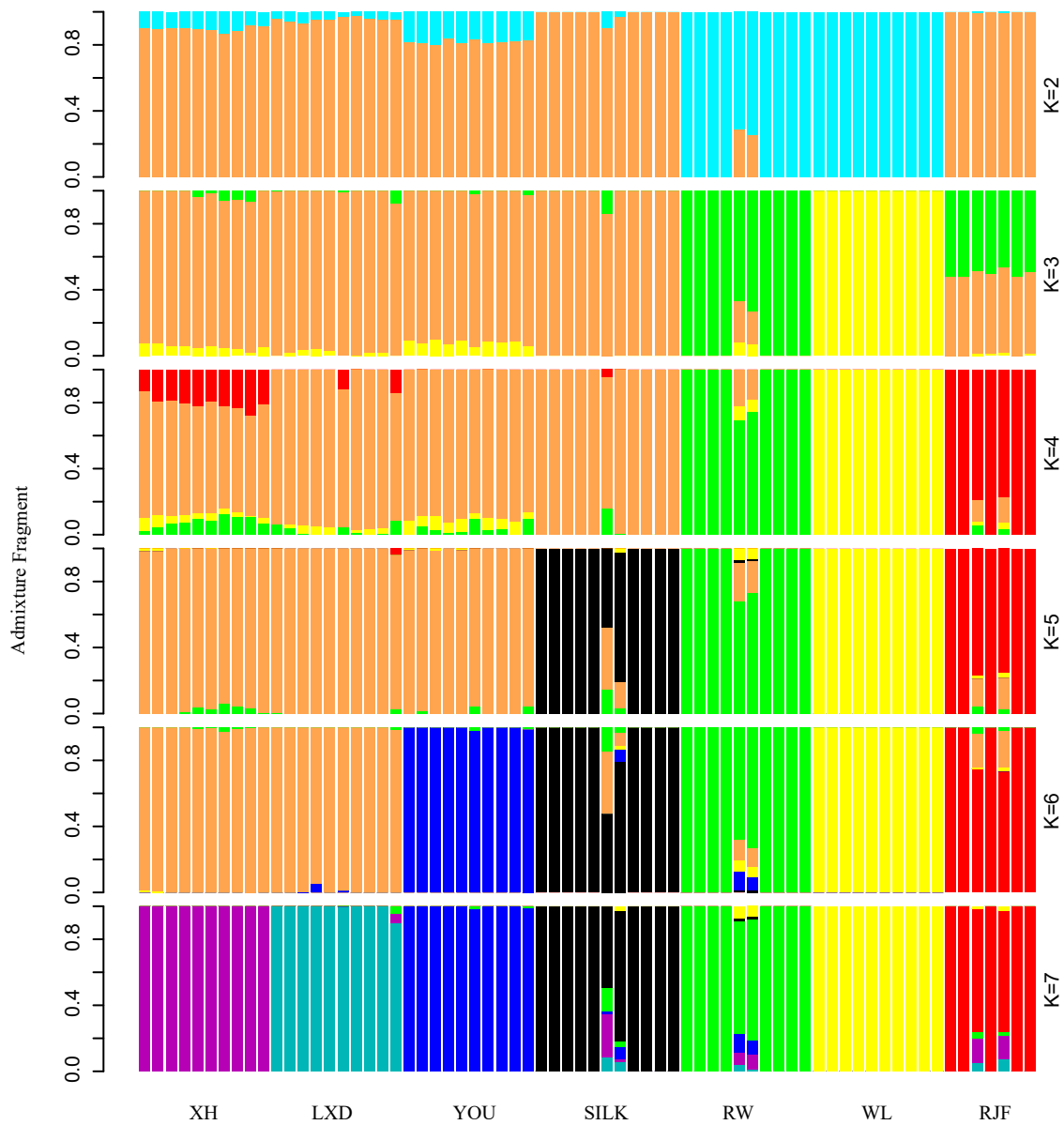

Figure S1

Supplement: Supplementary file 2 — Additional file 2: Figure S1. The inferred population stratification and individual genetic admixture in wild and domesticated chickens. A. The cross-validation procedure to infer the optimal number of genetic clusters (K) that presents the minimum cross-validation error. B. The delivered population stratification and individual genetic admixture with varying K (ranging from 2 to 7); colors in each column represent the individual ancestry proportions. XH: Guangdong Xinghua, LXD: Luxi Dou, YOU: Beijing You, SILK: Silkie, RW: Recessive White Rock, WL: White Leghorn, RJF: Red jungle fowls. [file 12864_2020_6613_MOESM2_ESM.pdf]

A

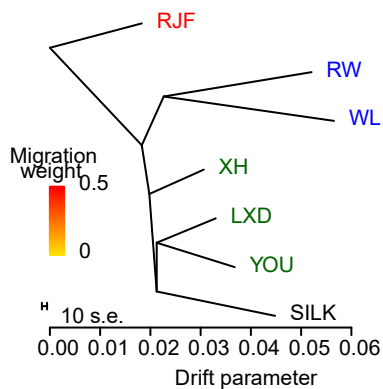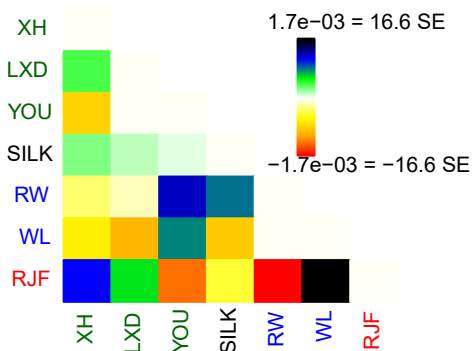

B

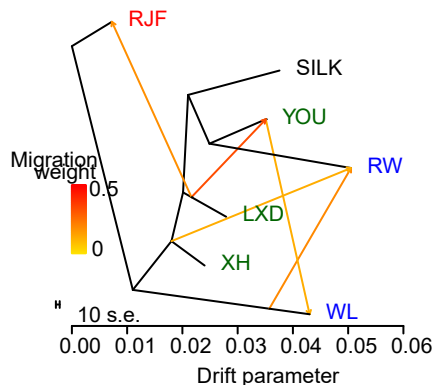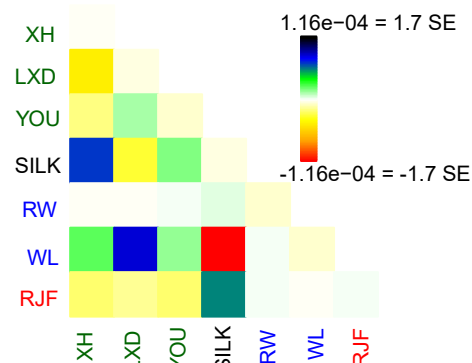

C

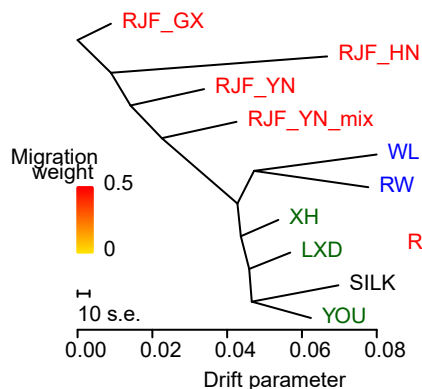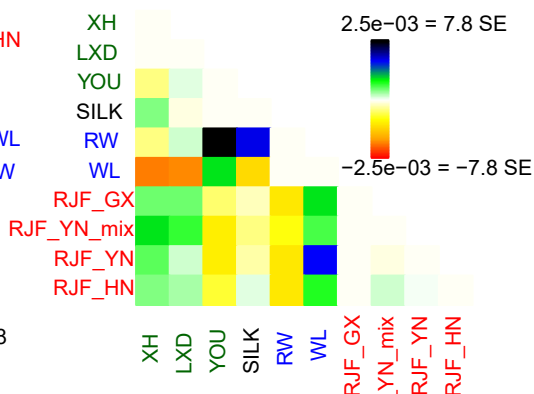

D

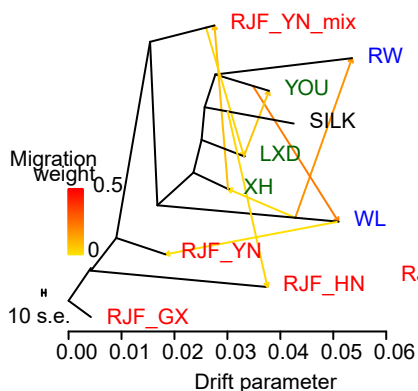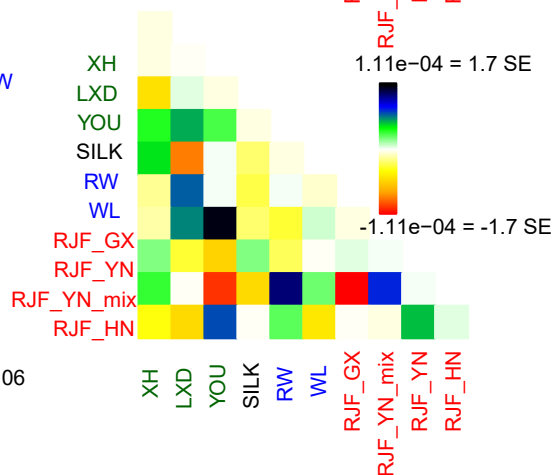

Supplement: Supplementary file 3 — Additional file 3: Figure S2. The deduced introgression events among chicken populations. RJFs are integrated into one outgroup in A and B, and are subdivided into distinct populations based on their geographic locations (RJF_HN from the Hainan province, RJF_GX from Guangxi, RJF_YN from Yunnan, among which, RJF_YN_mix indicates two admixed individuals) in C and D. A and C. (Left panel) The maximum likelihood tree (the breed symbol in red color represents red jungle fowls, blue: the commercial breeds, green: Chinese native breeds, black: the Silkie) and (Right panel) the residual matrix of the fitted model, where, the color in each cell [i, j] proportionally reflects the scaled residual covariance between population i and j, i.e. the residual covariance divides the average standard error (SE) of the observed covariances across pairs of population. The color scale bar is described in the palette on the right. Residuals above zero represent populations that are more closely related to each other in the data than in the fitted tree, and thus are candidates for introgression events. The fitted tree accounts for 87.43% of the variance in relatedness among populations in A, and 98.50% in C. B and D. (Left panel) The maximum likelihood tree with 5 and 8 deduced introgression events and the residual matrix of the improved model. The introgression events are highlighted as the arrows with colors from yellow to red, which represent the various weights of introgression. The arrow direction indicates the introgression direction. (Right panel) The residuals of the improved model are illustrated, where the color scale is the same as that in A. The fraction of the variance in relatedness among populations interpreted by the improved model rises up to 99.96% in B and 99.97% in D. [file 12864_2020_6613_MOESM3_ESM.pdf]

A

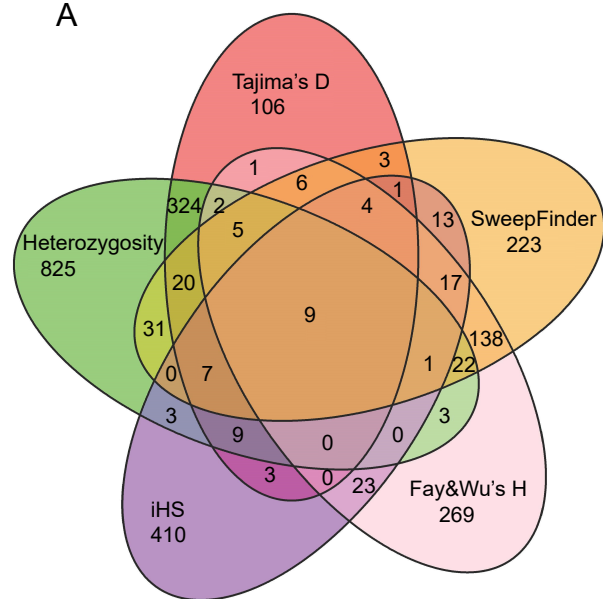

B

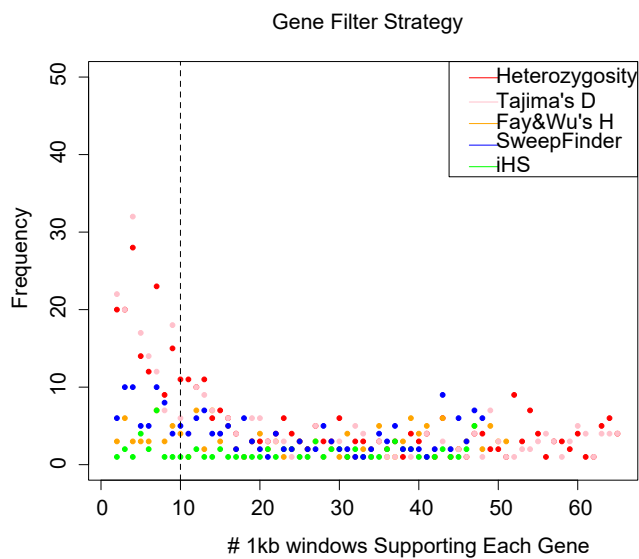

C

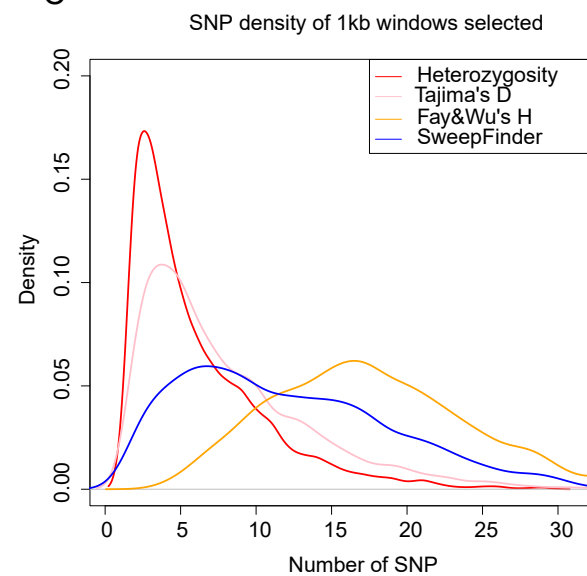

Figure S3

Supplement: Supplementary file 4 — Additional file 4: Figure S3. The selective sweeps derived from multiple statistics and the filtering strategy for the positively selected genes. A. Venn diagram of the identified selective sweeps from five methods including Heterozygosity, Tajima’s D, Fay and Wu’s H, integrated haplotype score (iHS) and SweepFinder. B. The density of SNPs with selection signals in 1-kb windows that locate in the identified selective sweeps and have 2% bottommost/topmost statistics for each method, reflecting the selection bias against smaller SNP number for Tajima’s D and Heterozygosity. C. The frequency of selected 1-kb windows supporting selective sweep genes for each method, determining the filter strategy of selective sweep genes as 10 1 kb-windows for Tajima’s D and Heterozygosity. [file 12864_2020_6613_MOESM4_ESM.pdf]

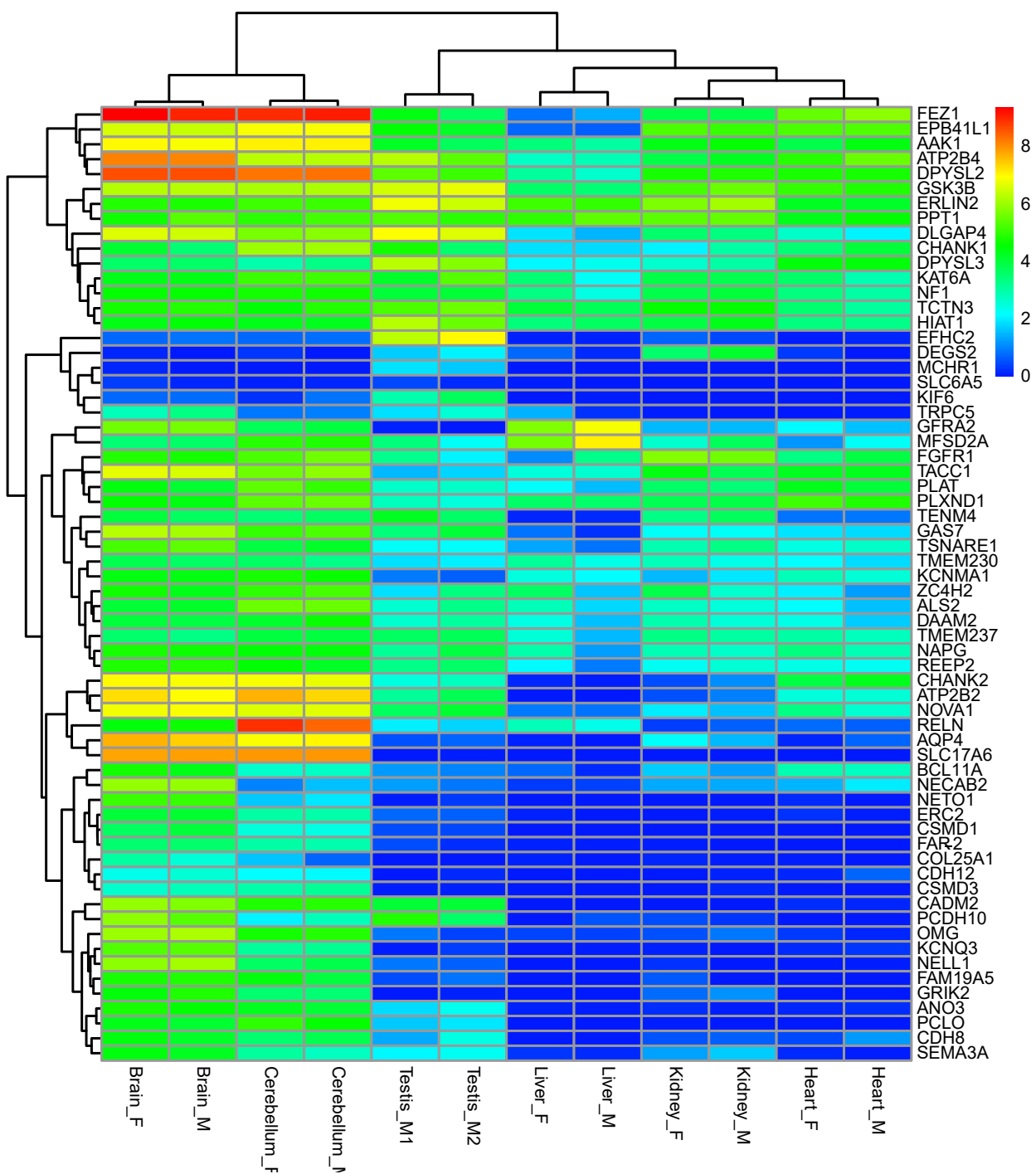

Figure S4

Supplement: Supplementary file 5 — Additional file 5: Figure S4. The expression pattern across 6 chicken tissues for the 65 selective sweep genes related to neurodevelopment. The gene expression pattern is based on the published RNA-Seq data of RJFs [31]. The gene expression level is calibrated on the logarithmic scale. The tissues include cerebrum, cerebellum, testis, liver, kidney and heart. For each tissue, one male and one female were examined, with the exception of testis where two males were examined. [file 12864_2020_6613_MOESM5_ESM.pdf]

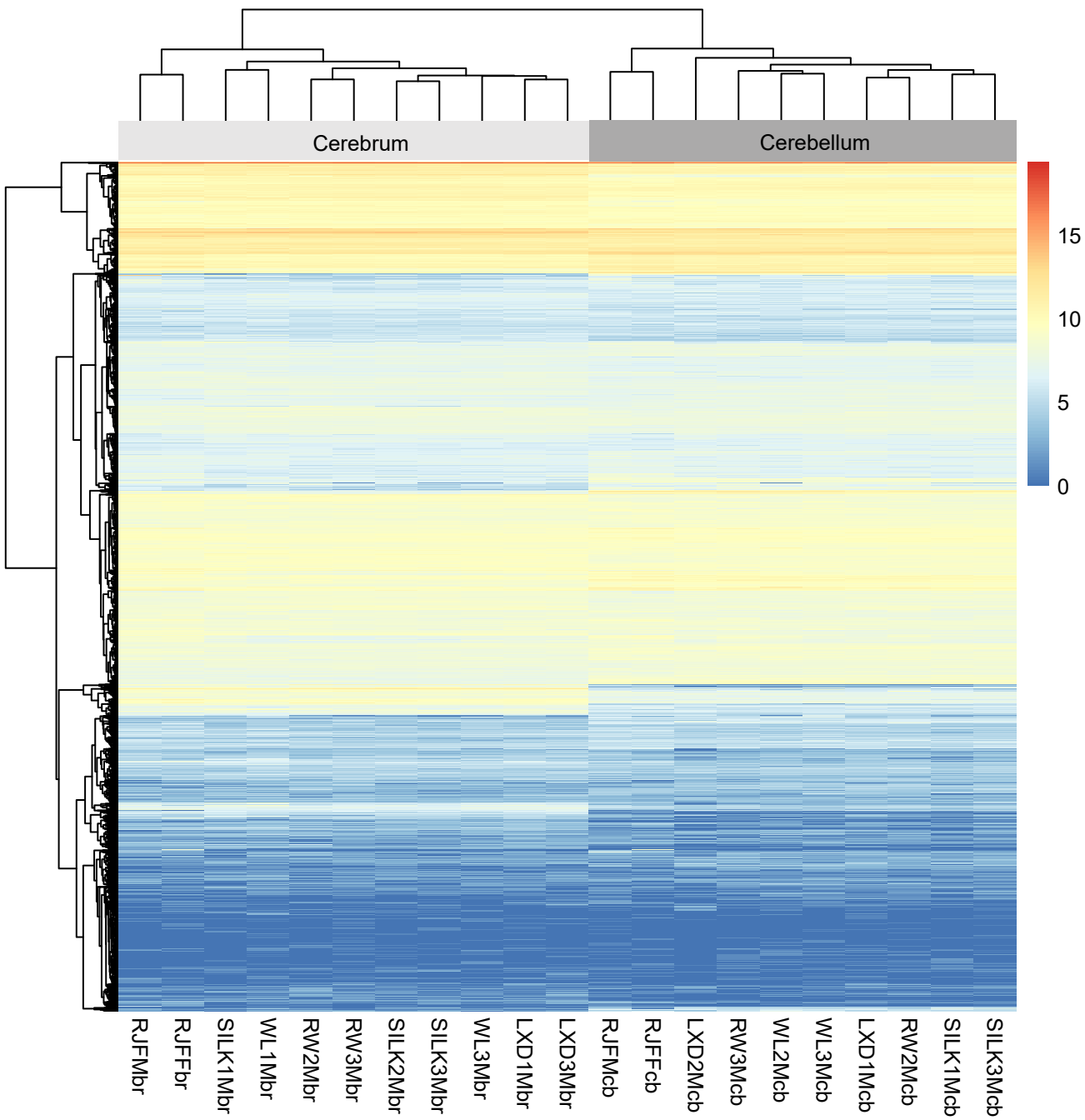

Figure S5

Supplement: Supplementary file 6 — Additional file 6: Figure S5. Clustering analyses of gene expression in cerebrum and cerebellum across chicken populations. [file 12864_2020_6613_MOESM6_ESM.pdf]

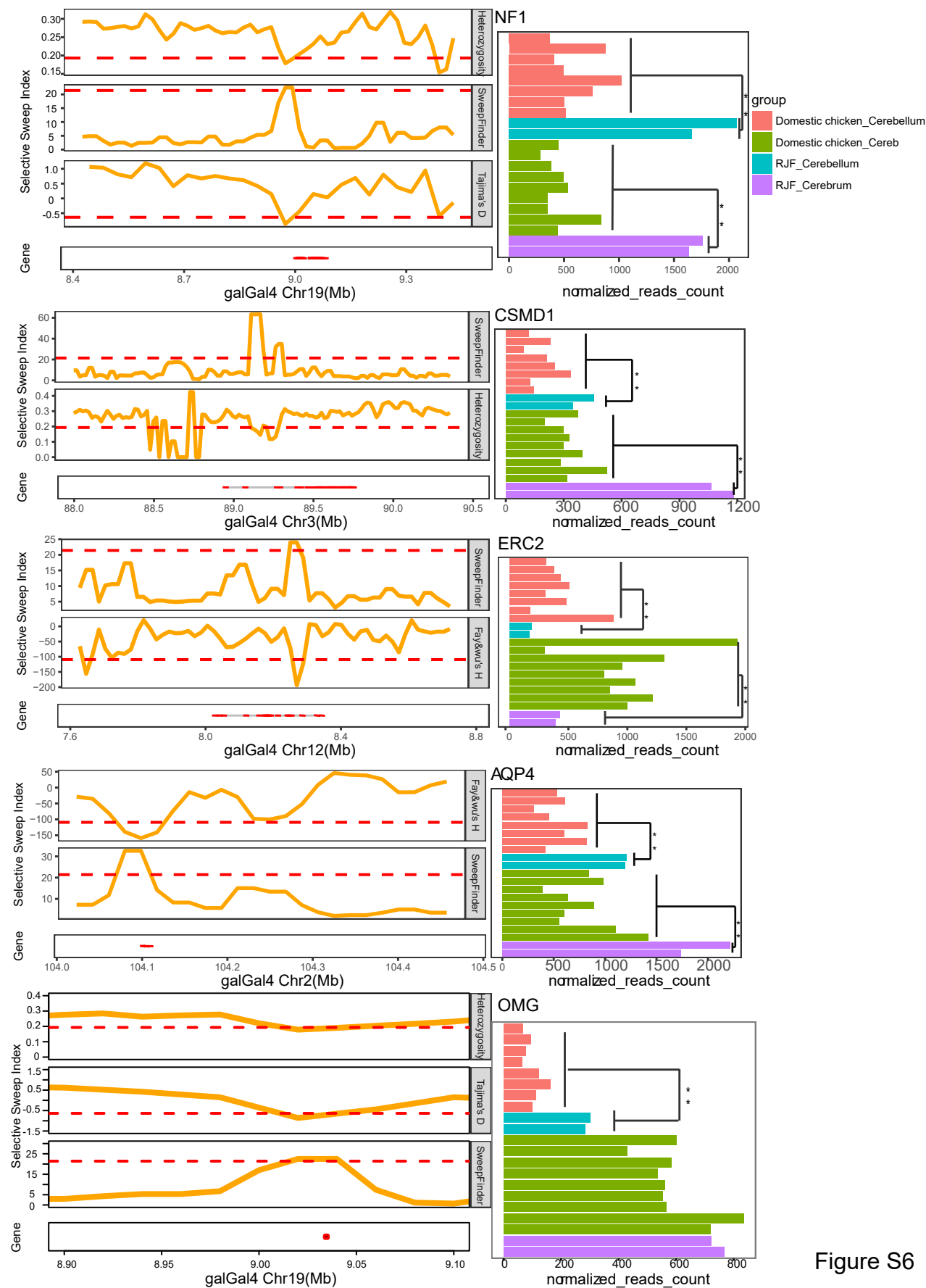

Supplement: Supplementary file 7 — Additional file 7: Figure S6. Selection signal and expression profiles of five representative genes associated with behavioral modification during chicken domestication. These genes are both positively selected genes and differentially expressed genes in cerebrum or cerebellum in domesticated chickens, whose functions in domestication-related behavioral alterations have been verified in mutant mouse models. The left panels indicate the statistics across different methods including Heterozygosity, Tajima’s D, Fay and Wu’s H, integrated haplotype score (iHS) and SweepFinder. The vertical axes represent the statistical values for each method; the horizontal axes represent the genomic coordinates around the target genes. The red dashed line in each method denotes the threshold above/below which signals were considered as positive selection. The right panels illustrate the gene expression levels of cerebrum and cerebellum in wild and domesticated chickens, where the double asterisks represent significant difference with a P value < 0.01. [file 12864_2020_6613_MOESM7_ESM.pdf]

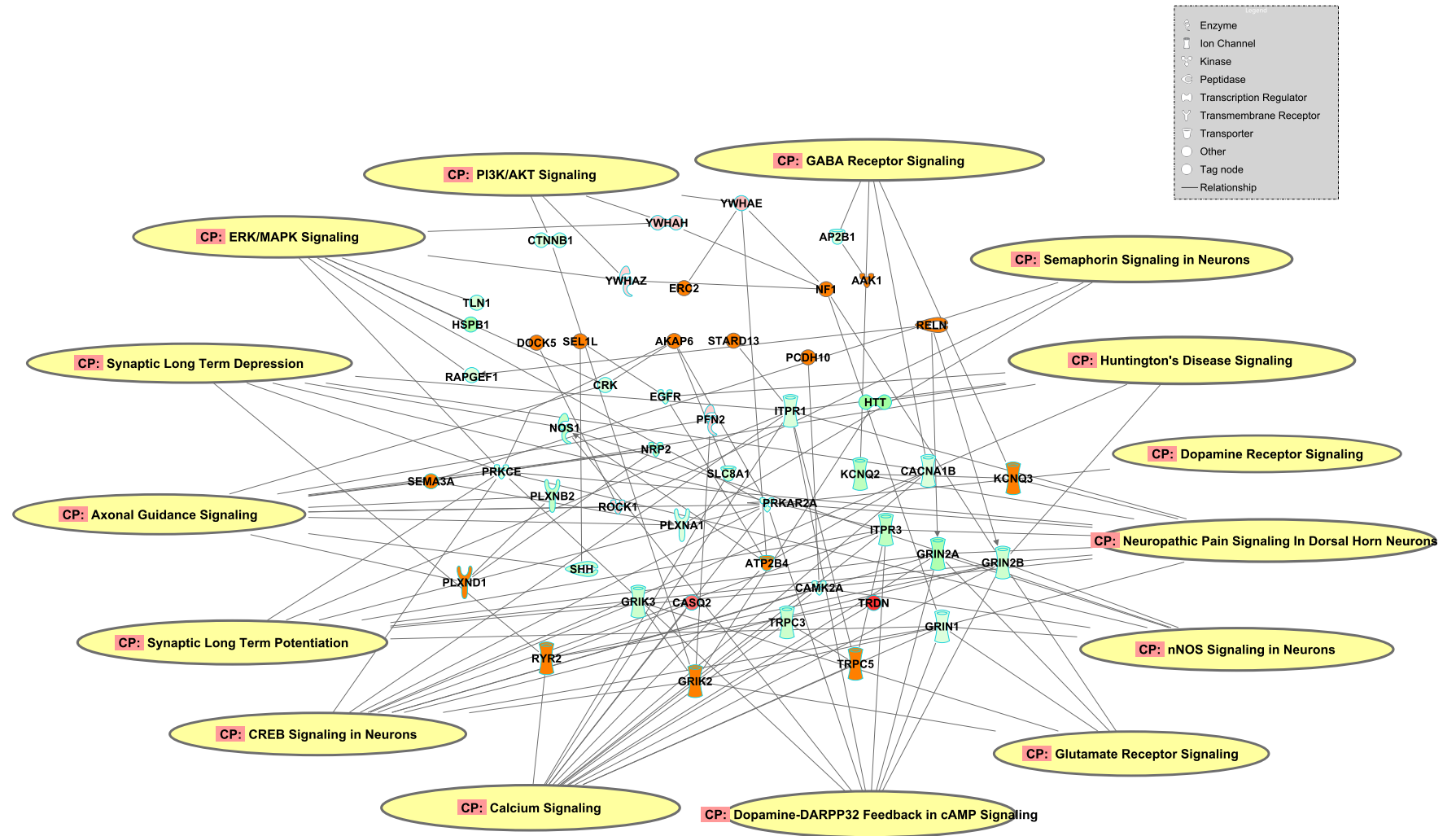

Figure S7

Supplement: Supplementary file 8 — Additional file 8: Figure S7. The regulatory network between the 53 positively selected genes (PSGs) and the differentially expressed genes (DEGs) of cerebrum and cerebellum in domesticated chickens compared with their wild counterparts. The molecules filled by orange color indicate the overlapped genes between PSGs and DEGs. The molecules filled by red or green color represent the up- or down-regulated DEGs, respectively, where the shades of filled colors represent the extent of the alteration at gene expression level. The solid lines imply direct interactions between molecules. The ovals filled by yellow color at outer layer indicate the canonical pathways related to neurological functions, in which, both PSGs and DEGs are known to participate. [file 12864_2020_6613_MOESM8_ESM.pdf]

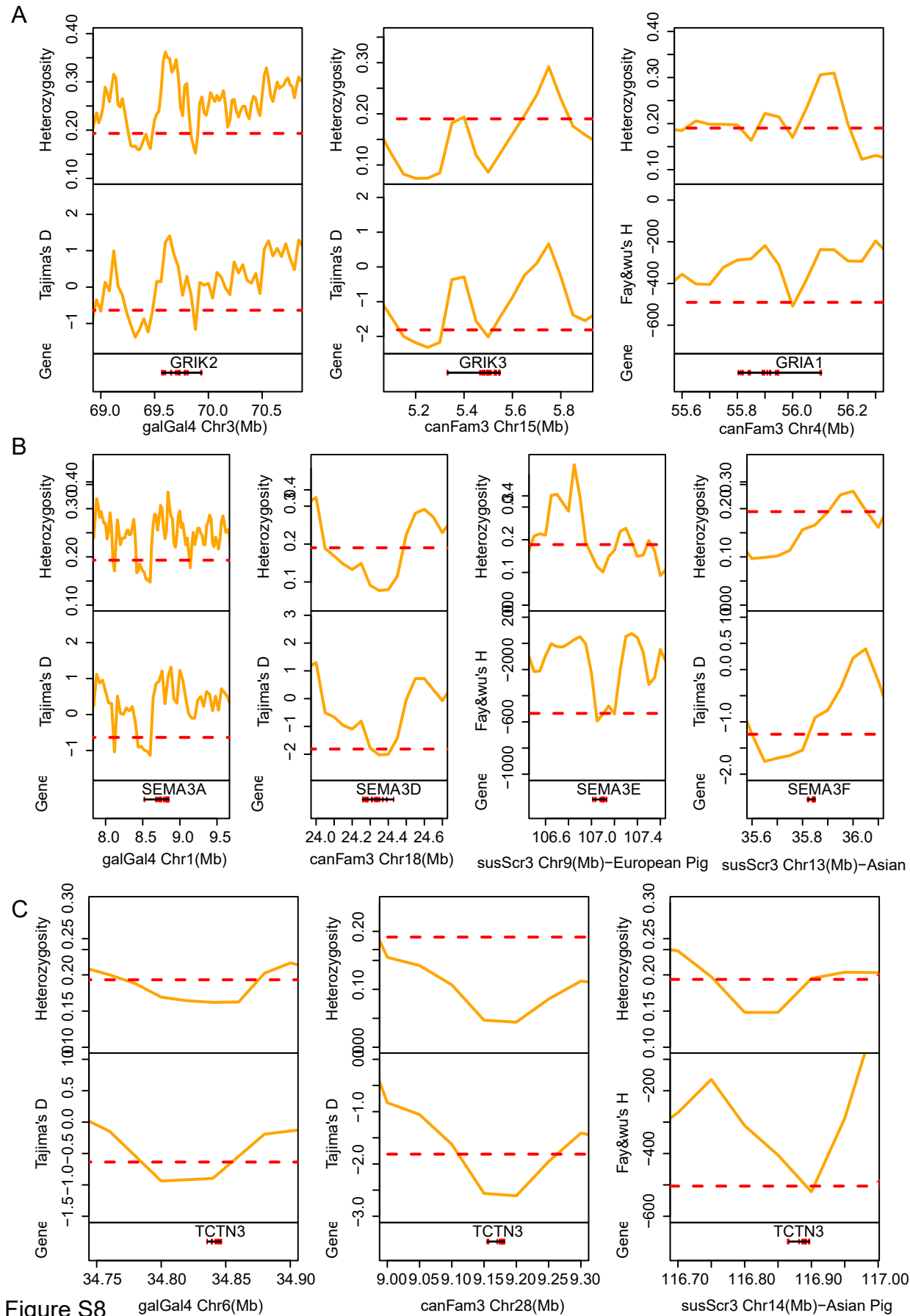

Figure S8

Supplement: Supplementary file 9 — Additional file 9: Figure S8. Selective signals on genes in convergent family of glutamate ionotropic receptors, semaphorins and tectonic proteins. The species include chicken (galGal), dog (canFam) and pig (susScr). The methods encompass Heterozygosity, Tajima’s D, and Fay and Wu’s H, integrated haplotype score (iHS) and SweepFinder. The vertical axes represent the statistical values for each method; the horizontal axes represent the genomic coordinates around the target genes. The red dashed line in each method denotes the threshold, above/below which signals were considered as positive selection. The red bars in genes indicate the exon regions of gene. [file 12864_2020_6613_MOESM9_ESM.pdf]

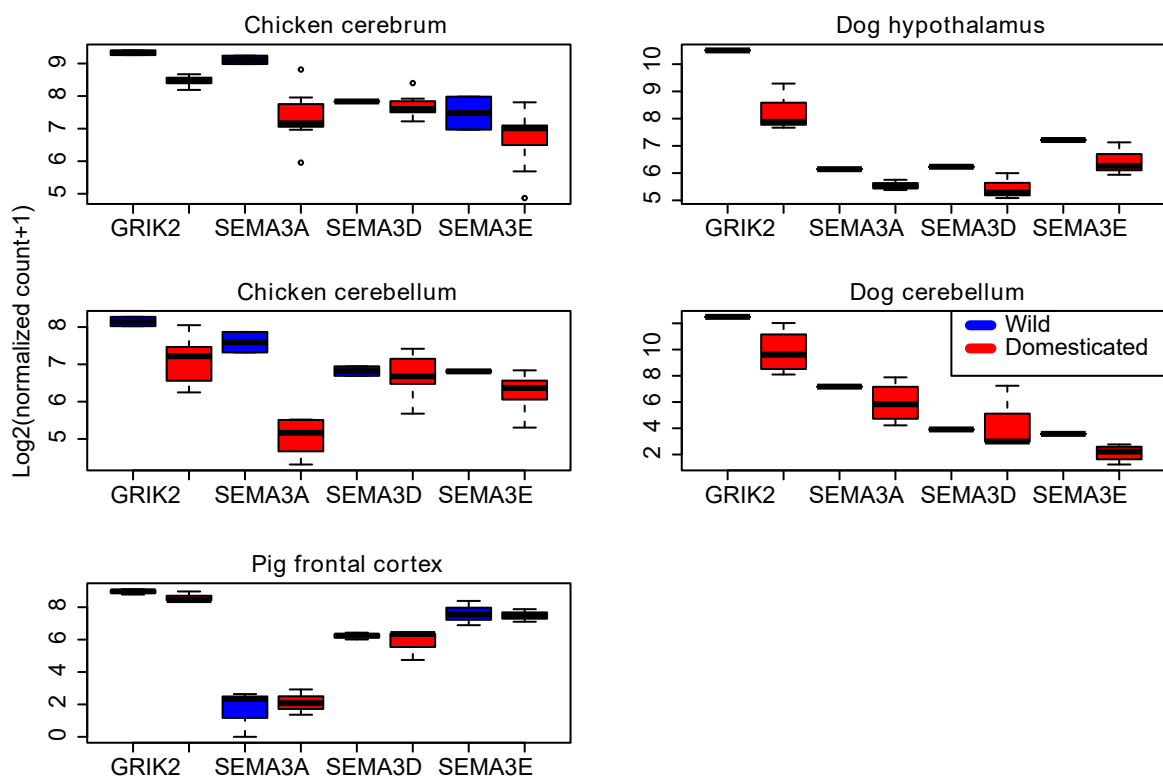

Figure S9

Supplement: Supplementary file 10 — Additional file 10: Figure S9. Expression level of genes in convergent family of glutamate ionotropic receptors, semaphorins and tectonic proteins in paired wild and domesticated animals. The species include chicken (galGal), dog (canFam) and pig (susScr). Brain tissues include cerebrum and cerebellum for chicken, frontal cortex for pig, and cerebellum and hypothalamus for dog. The vertical axes represent the gene expression levels; the horizontal axes indicate the genes in families of glutamate ionotropic receptors, semaphorins and tectonic proteins. [file 12864_2020_6613_MOESM10_ESM.pdf]

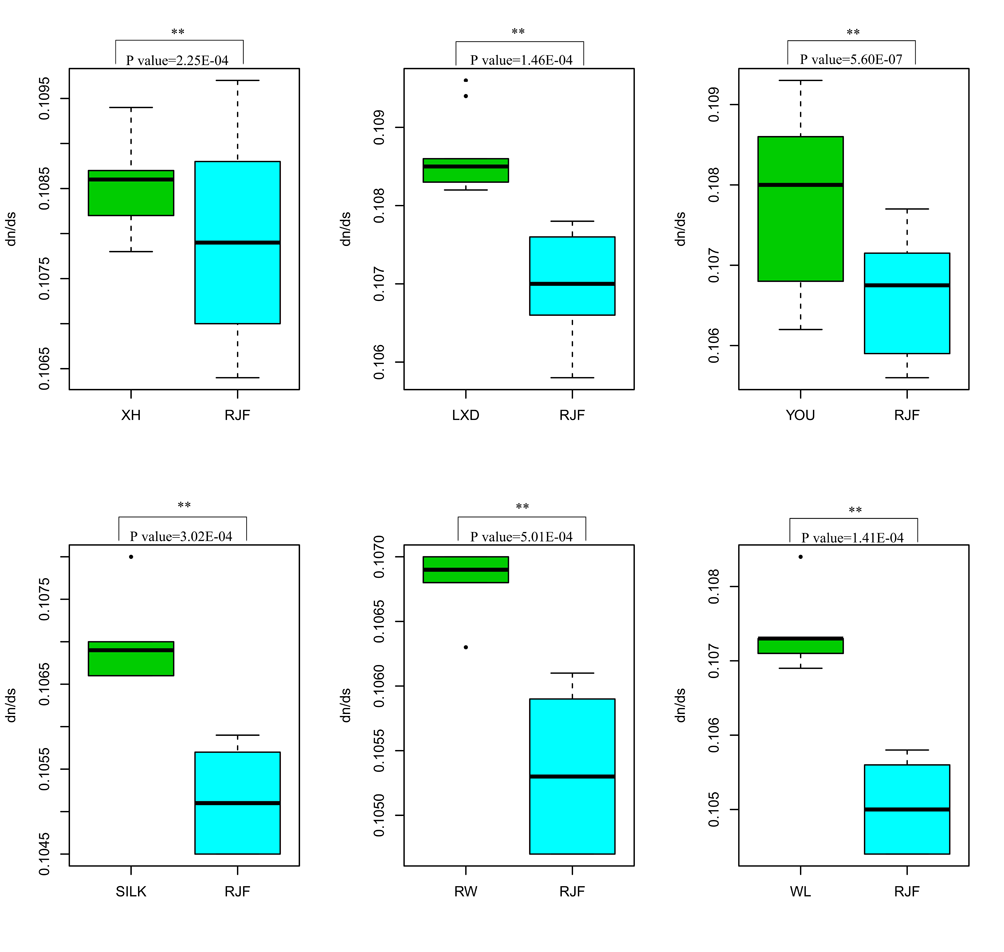

Supplement: Supplementary file 11 — Additional file 11: Figure S10. The comparison of nonsynonymous versus synonymous mutations (dN/dS ratio) between wild and domestic breeds across chicken (A), pig (B) and dog (C). Chicken population includes XH (Guangdong Xinghua), LXD (Luxi Dou), YOU (Beijing You), SILK (Silkie), RW (Recessive White Rock), WL (White Leghorn) and RJF (Red jungle fowls). One-way ANOVA is used for the significance test. [file 12864_2020_6613_MOESM11_ESM.tiff]

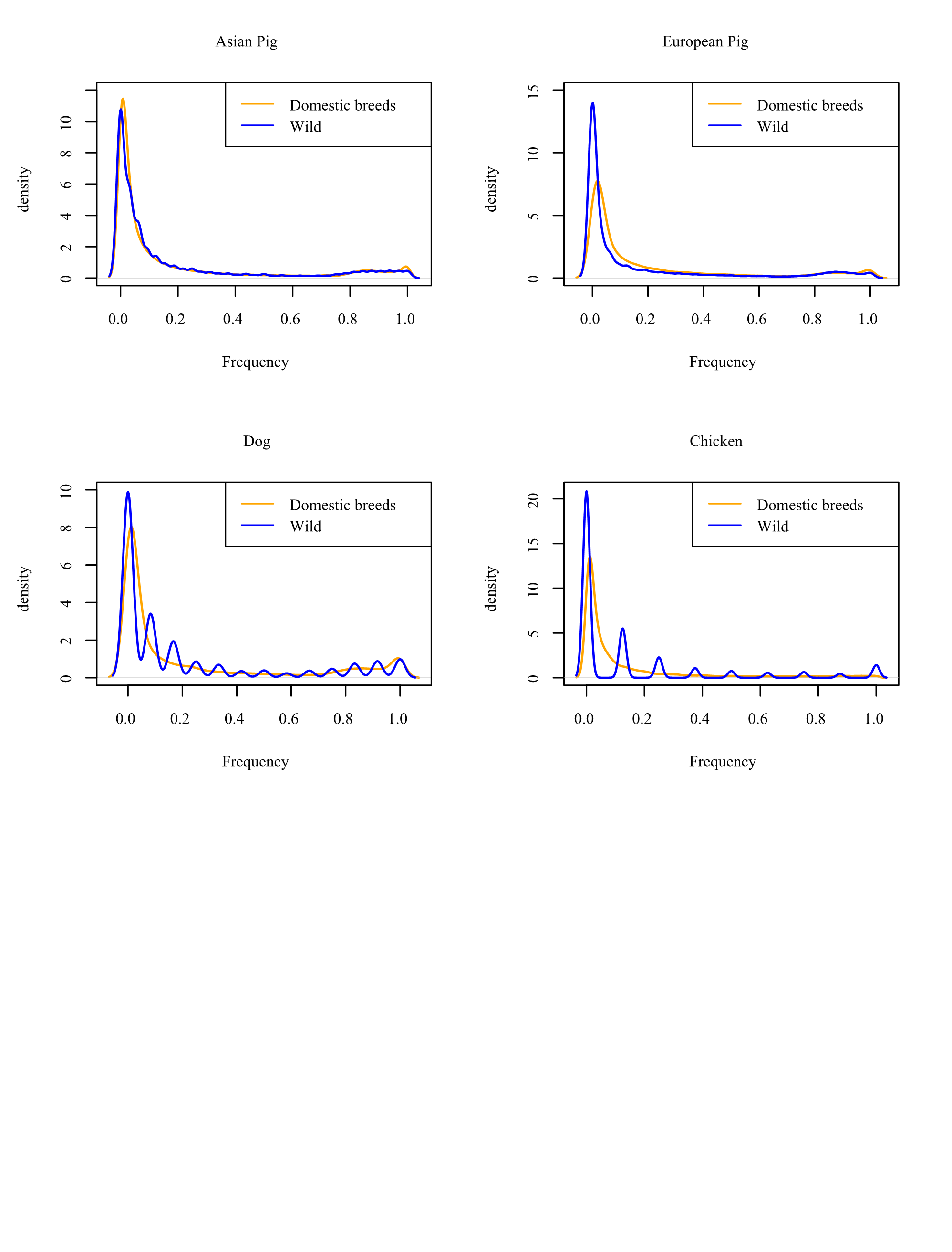

Supplement: Supplementary file 12 — Additional file 12: Figure S11. The comparison of frequency of deleterious variants between wild and domestic breeds across pig, dog and chicken. [file 12864_2020_6613_MOESM12_ESM.tiff]

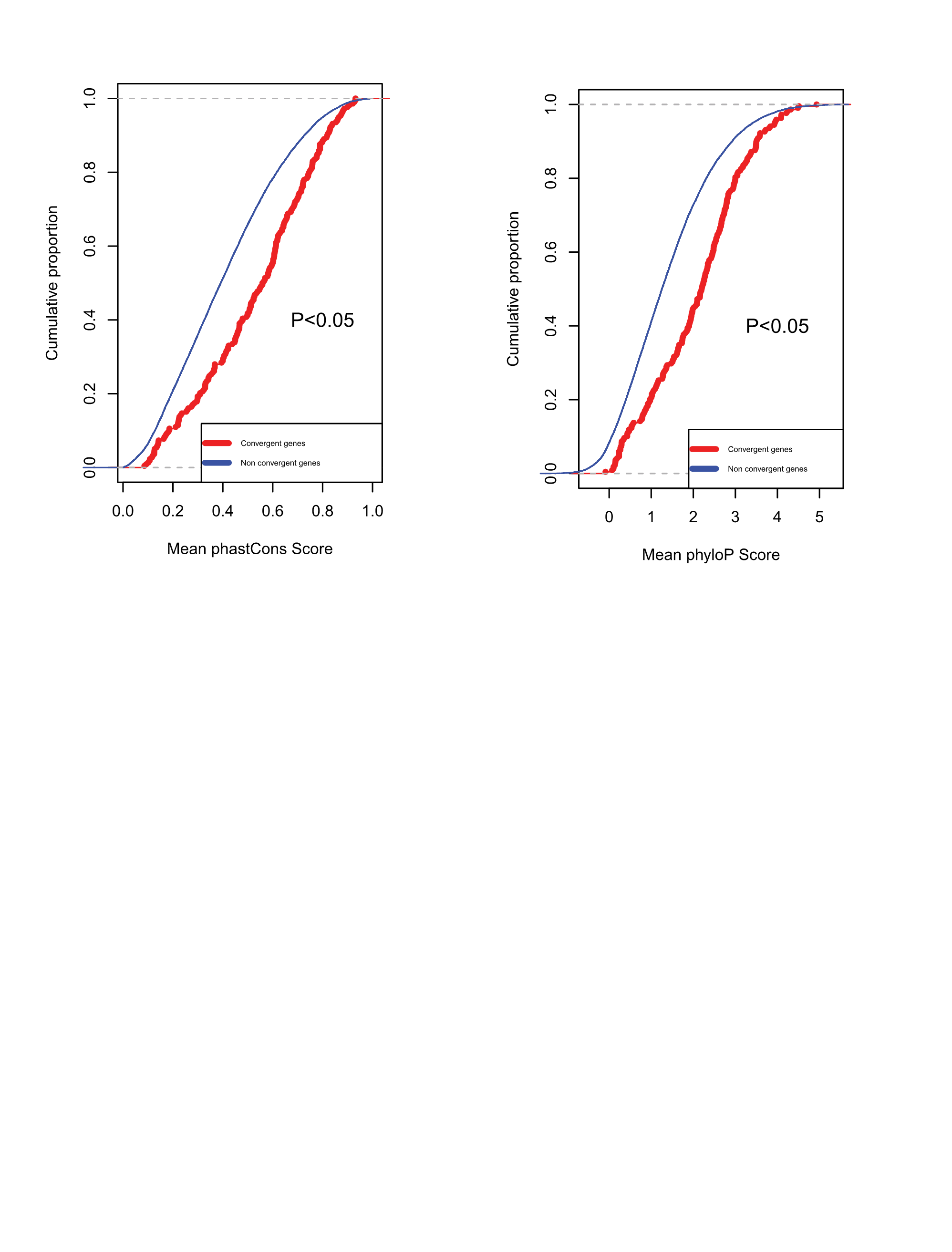

Supplement: Supplementary file 13 — Additional file 13: Figure S12. Conservation degree of the commonly targeted genes of selection. The cumulative distributions of phastCons and phyloP scores for the commonly targeted genes of selection (red) in comparison with other background genes (blue). Wilcoxon rank-sum test was implemented to check the significance of difference. [file 12864_2020_6613_MOESM13_ESM.tiff]

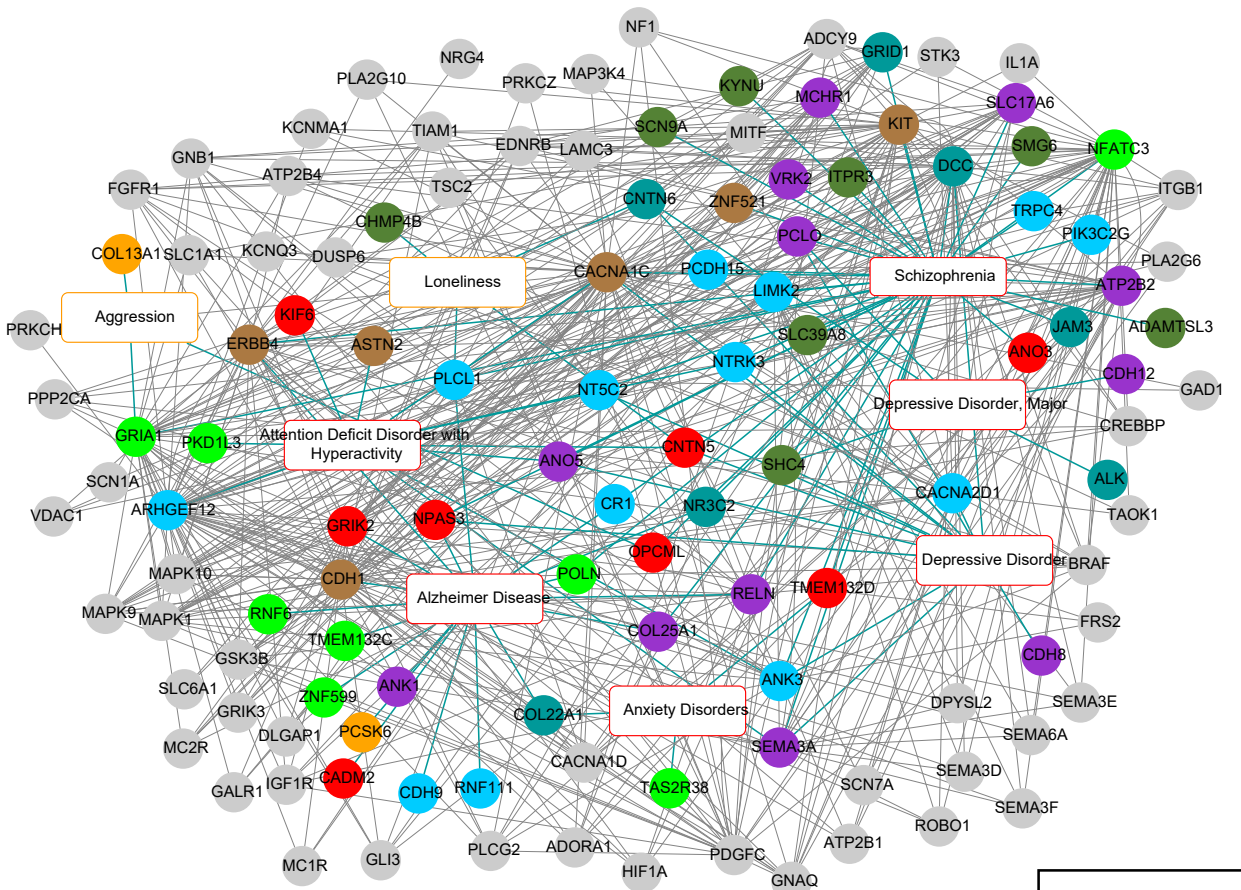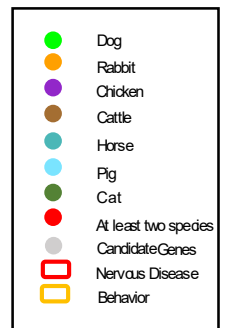

Supplement: Supplementary file 14 — Additional file 14: Figure S13. Comparison of the commonly selected genes in domesticated species with the risk factors of enriched human neurological diseases and behavioral disorders annotated in the PheGenI project. The commonly selected genes, were defined as those that showed signatures of selection in at least 2 domesticated species and were members in the common gene families and pathways that were shared by at least two species. The enriched neurological diseases and behavioral disorders in humans were anxiety, aggression, attention deficit hyperactivity, schizophrenia, depression and loneliness. The risk factors of these diseases were obtained from the PheGenI project. The conserved genes between the commonly selected genes during domestication and the risk factors of human neurological diseases and behavioral disorders were marked by circles and filled with different colors for different species. Red color indicates genes selected in at least 2 species. Besides of the conserved genes, other commonly selected genes may provide more risk factors (grey color) that may serve as novel candidates for these diseases, which were potentially related to the overrepresented neuropsychiatric diseases through functional interaction with the existed risk factors in the same pathways. [file 12864_2020_6613_MOESM14_ESM.pdf]

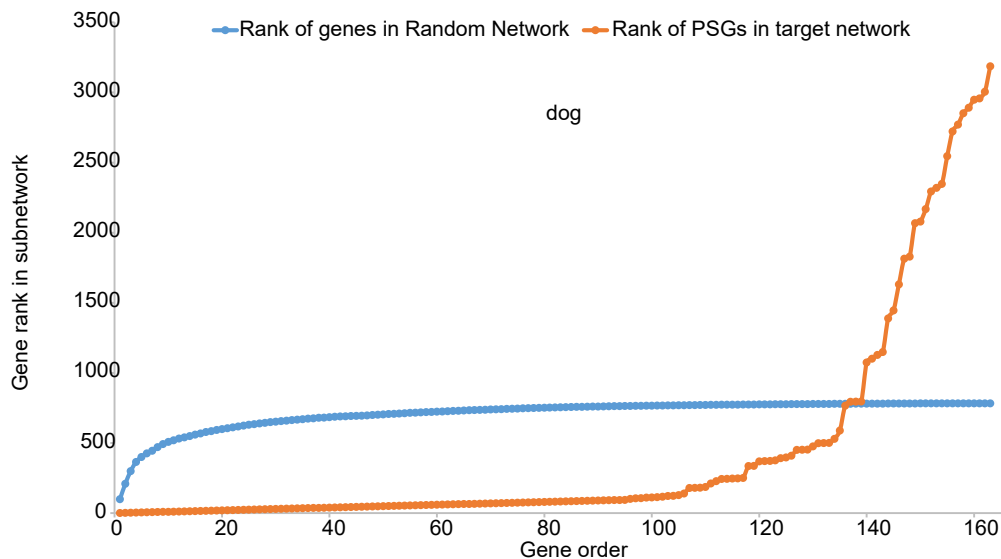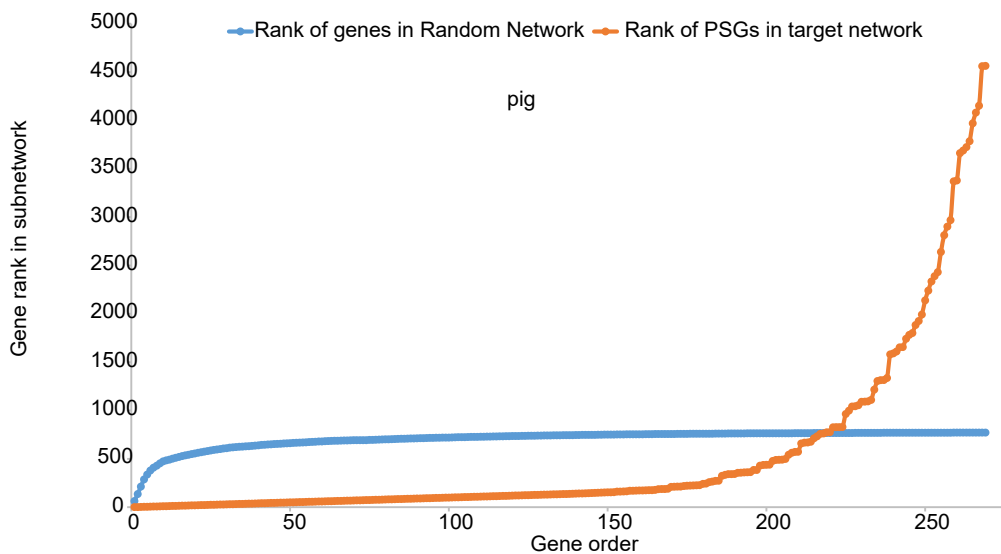

Supplement: Supplementary file 15 — Additional file 15: Figure S14. The rank of degree of the PSGs and the randomly selected genes from dog and pig gene-gene interaction network. The orange line is the rank of degree of the PSGs, calculated from their subnetwork and sorted by increasing x-coordinate. The blue line is the median rank of degree of randomly selected genes (Equal number with PSGs) in their subnetwork after 1000 times sampling. [file 12864_2020_6613_MOESM15_ESM.pdf]

Expected value  
Actual value

Chicken

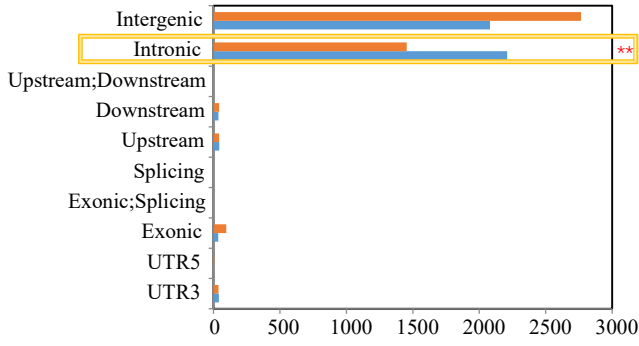

Dog

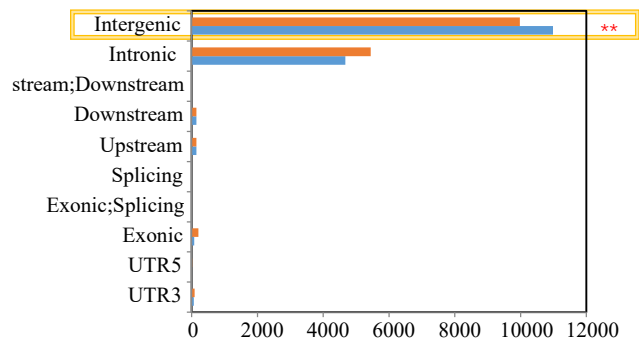

Pig\_Asian

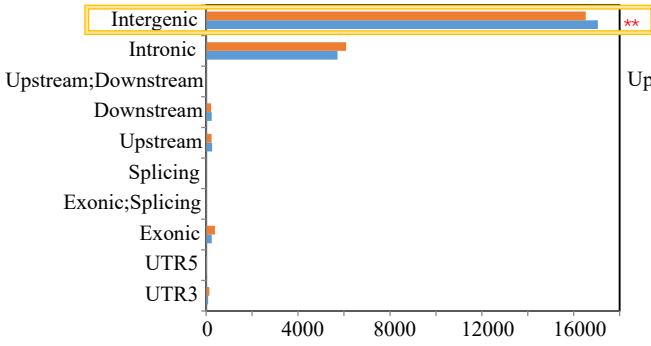

Pig\_European

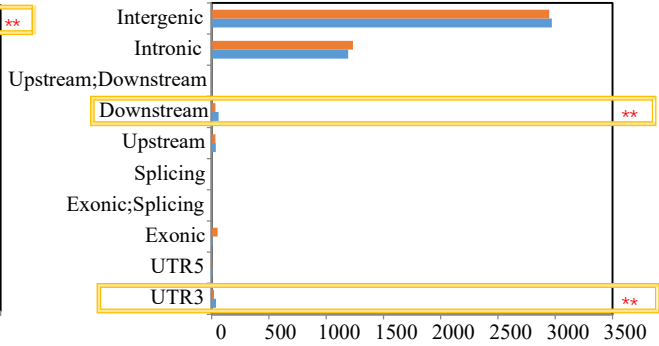

Supplement: Supplementary file 16 — Additional file 16: Figure S15. Enrichment of the putatively adaptive mutations in different genomic regions across pig, dog and chicken. The putatively adaptive mutations in response to domestication were defined as SNPs within selective sweeps with iHS < − 2 in domesticated animals. Genomic regions of exon, intron, upstream, downstream, UTR3, UTR5, splicing, and intergenic regions were considered. Upstream and downstream regions were defined as 1 kb upstream and downstream the transcription start and end site, respectively. The pigs in Asia and Europe were independently analyzed to account for their distinct origins. The significantly enriched genomic regions were circumscribed. Double asterisks represent significant difference with a P value < 0.01. [file 12864_2020_6613_MOESM16_ESM.pdf]
